# Psychological factors associated with postoperative cognitive outcomes in older adults: a systematic review and meta-analysis

**DOI:** 10.1016/j.bja.2026.01.031

**Published:** 2026-02-27

**Authors:** Anahita Amirpour, Lina Bergman, Jeanette Eckerblad, Gabriela Markovic, Ulrica Nilsson, Anna Falk

**Affiliations:** 1 Department of Neurobiology, Care Sciences and Society, Karolinska Institutet, Huddinge, Sweden; 2 Department of Clinical Sciences, Danderyd University Hospital, Karolinska Institutet, Stockholm, Sweden; 3 Division of Rehabilitation Medicine, Danderyd University Hospital, Stockholm, Sweden; 4 Perioperative Medicine and Intensive Care Function, Karolinska University Hospital, Stockholm, Sweden; 5 Department of Physiology and Pharmacology, Karolinska Institutet, Stockholm, Sweden

**Keywords:** anxiety, depression, meta-analysis, personality, postoperative cognitive complications, postoperative delirium, stress, systematic review

## Abstract

**Background:**

Older adults face increased risk of postoperative cognitive complications, which can prolong hospitalisation and impair function. Preoperative psychological factors may contribute to these cognitive outcomes, yet their impact remains unclear. This systematic review with meta-analysis synthesises evidence on associations between psychological factors and postoperative cognitive outcomes in older adults.

**Methods:**

A systematic review and meta-analysis, including synthesis without meta-analysis, of studies reporting preoperative psychological factors and postoperative cognitive outcomes in adults aged 60 yr or older was conducted. Five databases (Medline, Embase, Web of Science, PsycINFO, and CINAHL) were searched. The Newcastle Ottawa Scale or Cochrane Risk of Bias 2.0 tool was used for quality appraisal. Where feasible, random-effects meta-analyses were conducted alongside *P*-value synthesis and vote counting based on direction of effect.

**Results:**

Thirty studies (*n*=6714 subjects) were included. Four categories of psychological factors were identified: depression, anxiety, personality traits, and stress-related factors. Postoperative delirium was the most reported outcome, assessed in all studies with incidence ranging from 9% to 55.6%. Two studies assessed delayed neurocognitive recovery or postoperative neurocognitive disorder. In studies reporting effect estimates, the random-effects model showed a non-significant association between depression and postoperative delirium (odds ratio, 1.08; 95% confidence interval, 0.97–1.20). In the synthesis without meta-analysis, there was evidence of an association between the psychological factors and postoperative cognitive outcomes in 28 studies (*P*<0.001).

**Conclusions:**

Depression was the most consistently reported psychological risk factor for postoperative delirium in older adults, whereas evidence for anxiety, personality traits, and stress is limited. Multidimensional, standardised cognitive assessments are needed, and future studies should evaluate interventions to reduce risk and improve postoperative cognitive outcomes.

**Systematic review protocol:**

PROSPERO (CRD42024581115).

**Editor’s key points**

• Older adults face increased risk of postoperative cognitive complications, including delirium; however, the impact of preoperative psychological factors remains unclear.
• In this systematic review and meta-analysis, the authors synthesise evidence regarding psychological factors and postoperative cognitive outcomes in older adults, identifying depression as the most reported factor associated with delirium, with limited evidence for anxiety, personality traits, and stress-related factors.
• Future studies should use psychological instruments validated for older adults, predefine relevant covariates, and incorporate Comprehensive Geriatric Assessment for frail, older adults.

The number of older adults undergoing surgical procedures is increasing rapidly in Europe^1^ and North America,^2^ reflecting demographic shifts toward an ageing population. Older surgical patients face a distinct set of risks, particularly concerning cognitive complications caused by age-related brain atrophy, combined with diminished physiological resilience and multiple comorbidities.^3^ Postoperative cognitive complications include postoperative delirium (POD), delayed neurocognitive recovery (dNCR), and postoperative neurocognitive disorder (p-NCD), formerly referred to as postoperative cognitive dysfunction (POCD). These cognitive complications can impact consciousness levels, memory, attention, and executive functions^4^ and are associated with functional decline and reduced quality of life.^5^ The pooled incidence of POD is approximately 18%^6^ and reported rates of dNCR and p-NCD range from 2% to 36%.^7^ This wide variability reflects differences in study populations, cognitive instruments, cut-offs, and follow-up periods.^8^ Given their impact and the growing population at risk, identifying predisposing factors is crucial. Whereas previous research has focused largely on medical and surgical risk factors, a growing body of work explores the potential influence of psychological factors on postoperative outcomes.^9^, ^10^, ^11^, ^12^

Psychological factors comprise a heterogeneous set of constructs, including affective symptoms (e.g. anxiety), cognitive appraisal, stress reactivity, and personality traits.^13^ Although some studies have linked these factors to cognitive outcomes, the overall evidence remains inconclusive.^9^^,^^10^ For example, specific personality traits have been associated with POD,^11^ and self-efficacy, pessimism, and emotional distress have been linked to postoperative recovery trajectories.^12^ However, evidence on the association between psychological factors and dNCR/p-NCD remains limited.

Previous systematic reviews have predominantly focused on anxiety or depression in relation to POD, often excluding other psychological factors and not addressing dNCR or p-NCD as outcomes.^14^, ^15^, ^16^ Moreover, their generalisability to older adults is limited. Most reviews include mixed-age populations or restrict their focus to cardiac surgery, with occasional subgroup analyses by age.^14^, ^15^, ^16^, ^17^

Compounding these gaps, psychological symptoms in older adults are frequently underrecognised. Coexisting somatic comorbidities may obscure psychological complaints,^18^ and individuals may hesitate to disclose concerns to healthcare providers because of stigma or uncertainty about relevance.^19^ These barriers suggest that psychological factors remain insufficiently explored as contributors to postoperative cognitive outcomes in older adults.

This systematic review and meta-analysis, including synthesis without meta-analysis (SWiM), aims to address these gaps. We hypothesise that preoperative psychological factors are associated with an increased risk of POD, dNCR, or p-NCD in older adults. We aim to synthesise current evidence on these associations, evaluate the methodological quality of existing studies, and consider clinical implications for preoperative risk stratification and future research directions.

## Methods

A systematic review with meta-analysis and SWiM was conducted.^20^ We registered our study protocol in PROSPERO (ID: CRD42024581115) and followed the Preferred Reporting Items for Systematic Reviews and Meta-Analyses (PRISMA) reporting guidelines.^21^

### Search strategy

We conducted a literature search in the following databases: Medline (Ovid), Embase, Web of Science (Clarivate Analytics), PsycINFO (EBSCOhost), and CINAHL (EBSCOhost). The initial literature search was conducted on October 1, 2024, and the search strategy was developed in Medline (Ovid) in collaboration with librarians at the Karolinska Institutet University Library. For each search concept, Medical Subject Headings (MeSH) terms and free text terms were identified. The search was then translated, in part using Polyglot Search Translator, into the other databases. No language restriction was applied; however, only articles in English were eligible for inclusion. Databases were searched from inception, and the strategies were peer reviewed by another librarian before execution. Deduplication was done using Covidence (Veritas Health Innovation, Melbourne, VIC, Australia). The literature search was last updated on May 27, 2025, by rerunning the searches and deduplicating against previous results using Covidence. Full search strategies for all databases are available in the Supplementary material.

### Data selection and screening

We included studies with a pre- and post-surgery design, both retrospective and prospective cohort studies, and RCTs reporting secondary outcomes fulfilling this review’s aims. Psychological factors were defined as emotional, mood-related, or personality-related characteristics, such as depressive symptoms and neuroticism. These were assessed using either patient-reported outcome measurements or clinician-administered instruments. Cognitive outcomes were evaluated using cognitive screening tools or formal neuropsychological tests.

Four authors (AA, AF, UN, LB) independently screened the first literature search with abstracts and full text using Covidence by a double screening approach. We resolved any screening conflicts through discussion or, if needed, with a third co-author. The full search strategies are presented in Supplementary Table 1.

### Eligibility criteria

Our inclusion criteria were (i) quantitative studies performed on adults ≥60 yr undergoing surgery and (ii) reported group comparison data on psychological factors for exposed and non-exposed groups, or associations (effect sizes, *P*-values) between the possible psychological factors and the cognitive outcomes such as POD, dNCR, and p-NCD.

We excluded non-quantitative research, studies involving patients undergoing cataract surgery, Parkinson's disease-related surgery (such as deep brain stimulation), epilepsy surgery, studies with mixed-aged groups, children or adolescents, reviews, conference abstracts, case reports, and dissertations. Studies that did not specify type of postoperative cognitive complication and its association with psychological factors were excluded. Excluded from inclusion were also studies defined as high risk of bias in the quality appraisal. After the initial search, we decided to include studies published from 2014 to 2024; this timeframe was primarily chosen for feasibility and to focus on studies reflecting contemporary perioperative care and delirium assessment practices.

### Data extraction

Two authors (AA, AF) piloted the data extraction form and then independently extracted data in Covidence and later into Microsoft Excel™ (Microsoft Corp., Redmond, WA, USA). We held weekly meetings to discuss any uncertainties during data extraction and make amendments to the form. For each study, we extracted the following variables: (i) study characteristics: study design and setting (author, location, sample size); (ii) patient characteristics (age, sex, educational background); (iii) perioperative factors (type of surgery, type of anaesthesia); (iv) psychological factor (type of factor, assessment instrument); (v) cognitive assessment and outcome (type of instrument, type of outcome, timing of assessment); and (vi) statistical results (effect sizes, *P*-values, group comparisons). The type of surgery was categorised as cancer, cardiac, orthopaedic, urologic, gynaecologic, or mixed procedures. Where study authors had not reported statistical results (two studies), we e-mailed the authors but did not receive a response.

### Quality appraisal and risk of bias

Three authors (AA, AF, UN) performed independent quality appraisal for the included articles using the Newcastle Ottawa Scale (NOS) (score range, 0–9) for observational studies.^22^ A score of ≤3 was considered high risk of bias, scores between 4 and 6 were considered medium risk of bias, and a score of ≥7 was considered low risk of bias. We used Cochrane’s Risk of Bias 2.0 tool (Cochrane, London, United Kingdom)^23^ to assess the experimental studies. When two authors had conflicting ratings on an article, the third researcher was assigned to also perform the quality appraisal.

### Data synthesis and analysis

We tabulated and summarised quality appraisal, study and patient characteristics, and findings descriptively. The tables were ordered by the quality appraisal ratings. We conducted two separate meta-analyses: one for studies reporting multivariate adjusted estimates and another for studies reporting univariate estimates. We used odds ratios (ORs) (adjusted or unadjusted) with corresponding 95% confidence intervals (95% CIs) as the effect size and precision. We present the meta-analyses using forest plots.

Because all studies did not report effect sizes, we applied alternative synthesis methods including compiling *P*-values, with Fisher’s method^24^ and vote counting based on direction of effect,^24^^,^^25^ each supported by visual displays. For studies reporting *P*-values, we used an albatross plot to see the rough magnitude and direction of associations.^20^^,^^26^ We created an effect direction plot following the method described by Boon and Thomson^25^ to see the direction of associations for the studies, including those that did not report *P*-values. We grouped studies according to the investigated psychological factors; categorised the effect as positive, negative, or mixed/conflicting; and represented them using directional arrows.

We assessed statistical heterogeneity with an *I*^2^-test, with values >50% indicating moderate heterogeneity. To explore potential publication bias and identify outliers in the multivariate meta-analysis, we performed Egger’s test using a weighted regression model, presented in a funnel plot.^27^ The statistical analysis and plots were performed in R (R Foundation for Statistical Computing, Vienna, Austria) and Microsoft Excel. The R packages used were library forestplot; ggplot2; readxl; metap. All tests were two sided, and statistical significance was set at α <0.05.

### Patient and public involvement

Patient or public involvement was not included in this systematic review.

## Results

### Study selection

**Fig 1:**
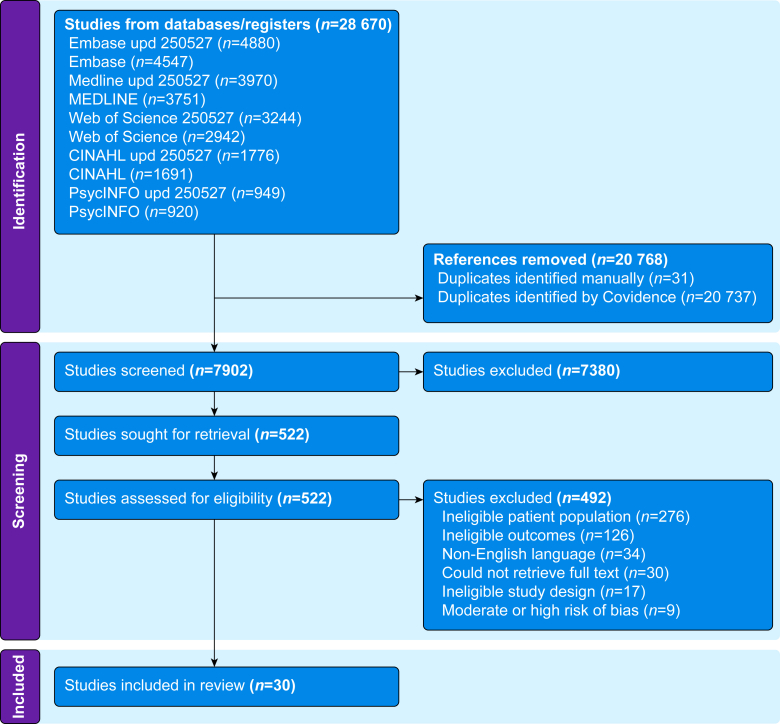
Preferred Systems Reporting Items for Systematic Reviews and Meta-analyses flow diagram. Upd 250527, Updated literature search Embase 250527.

The database search resulted in 28 670 articles. After deduplication, a total of 7902 articles were screened by title and abstract, of which 7380 were excluded. This left 522 studies for full-text assessment. Of these, 492 were excluded; the most common reason was ineligible patient population. In total, 30 articles met our inclusion criteria and were included in the data extraction (Fig. 1).

### Quality appraisal and risk of bias

Only studies with low risk of bias were included in the systematic review. However, three studies^28^, ^29^, ^30^ reported effect sizes inaccurately. They conducted logistic regression analyses but misreported the results as risk ratios^29^ or hazard ratios^28^^,^^30^ instead of ORs. Despite these reporting errors, the studies were deemed eligible for inclusion in the meta-analysis.

### Meta-analysis and synthesis

**Fig 2:**
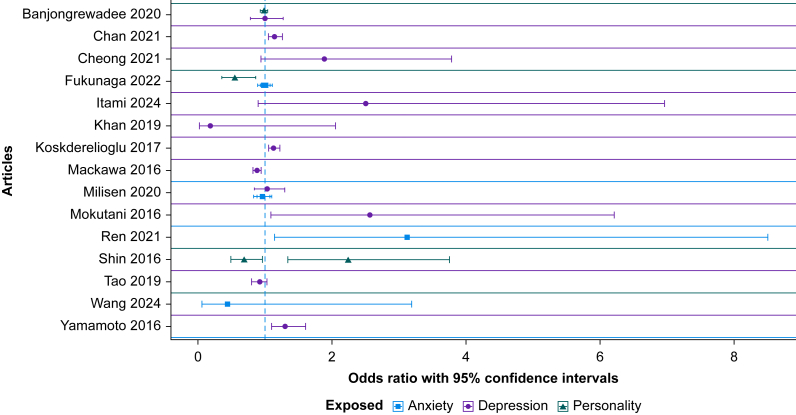
Forest plot of studies reporting multivariate data. The y-axis lists individual studies; the x-axis the odds ratios with 95% confidence intervals. The postoperative cognitive outcome is delirium in these studies. Colours indicate different psychological factors (blue=anxiety, purple=depression, green=personality). Some studies reported multiple exposures, which explains repeated data points.

**Fig 3:**
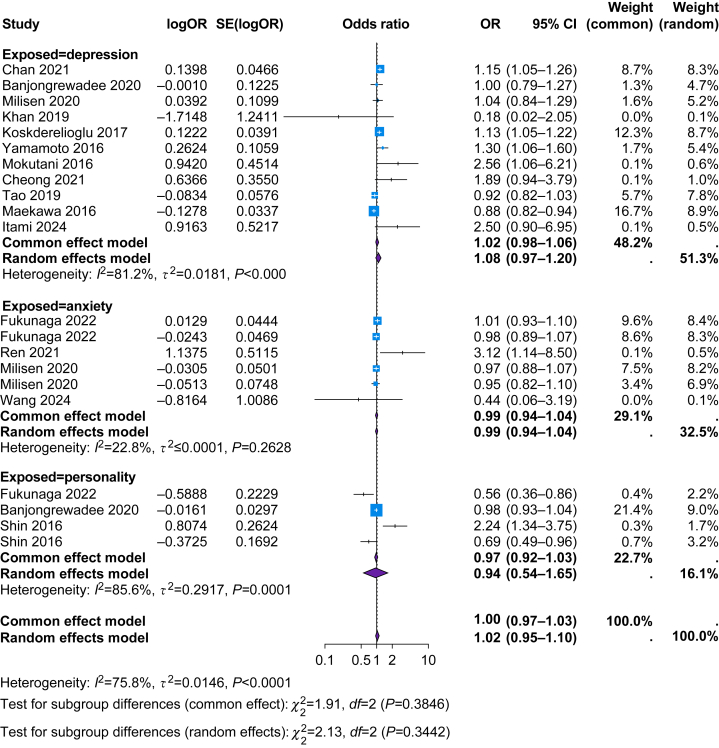
Random-effects model of studies reporting multivariate data. 95% CI, 95% confidence interval.

**Fig 4:**
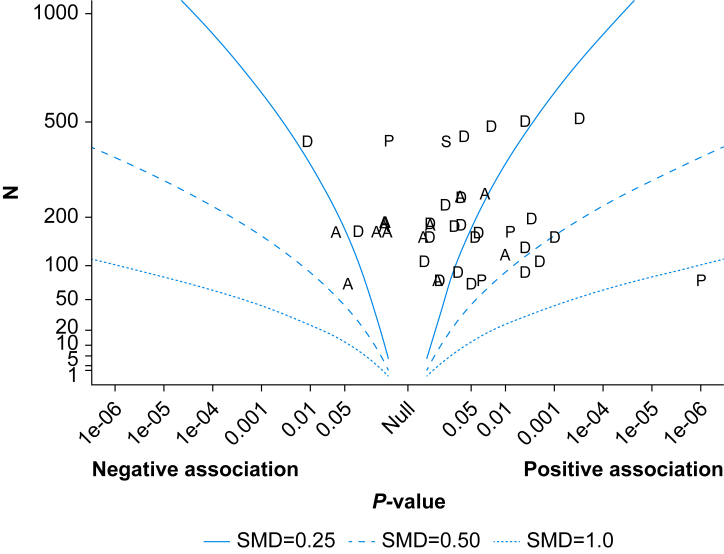
Albatross plot of studies reporting *P*-values. The y-axis reflects the sample sizes in the original studies; x-axis indicates the direction of effect (negative or positive). Psychological factors are letter coded: A=anxiety, D=depression, P=personality, S=stress-related factor. Each letter represents a study that assessed a psychological factor. All associations should be interpreted as approximate. SMD, standardized mean difference.

Two separate random-effects models were applied using data from 18 studies: 15 studies reported multivariate data^28^, ^29^, ^30^, ^31^, ^32^, ^33^, ^34^, ^35^, ^36^, ^37^, ^38^, ^39^, ^40^, ^41^, ^42^ (Figs 2 and 3) and three reported univariate data^29^^,^^43^^,^^44^ (Supplementary Figs 1 and 2). In total, 28 studies reported *P*-values and were included in the *P*-value synthesis^28^, ^29^, ^30^, ^31^, ^32^, ^33^, ^34^, ^35^, ^36^, ^37^, ^38^, ^39^, ^40^, ^41^, ^42^, ^43^, ^44^, ^45^, ^46^, ^47^, ^48^, ^49^, ^50^, ^51^, ^52^, ^53^, ^54^, ^55^ (Fig. 4). All 30 studies were included in the vote counting based on direction of effect which is presented in Supplementary Table 4.

### Study characteristics

Among the included studies, most were observational in design, either as prospective cohort studies^28^, ^29^, ^30^, ^31^, ^32^^,^^35^, ^36^, ^37^, ^38^, ^39^, ^40^, ^41^, ^42^, ^43^, ^44^, ^45^, ^46^, ^47^, ^48^^,^^50^, ^51^, ^52^, ^53^, ^54^^,^^56^^,^^57^ or retrospective.^33^ Three RCTs were also included^34^^,^^49^^,^^55^ (Table 1). Sample sizes (*n*) ranged from 72 to 517, with a total of 6714 across all studies. Sixteen countries were represented, and three were multicentre trials spanning several countries.^39^^,^^46^^,^^56^ The majority of studies were conducted in Asia,^28^, ^29^, ^30^, ^31^, ^32^, ^33^^,^^35^^,^^37^^,^^38^^,^^40^, ^41^, ^42^^,^^45^^,^^48^^,^^51^, ^52^, ^53^, followed by North America,^36^^,^^43^^,^^44^^,^^47^^,^^50^^,^^54^^,^^55^ Europe,^34^^,^^46^^,^^49^^,^^56^^,^^57^ and Africa.^39^

**Table 1:** Characteristics of included studies. Data presented as counts, percentages, mean (sd), or median (IQR) when reported. APAIS-A, Amsterdam Preoperative Anxiety and Information Scale—Anxiety; APAIS-NFA, Amsterdam Preoperative Anxiety and Information Scale—Need for Information; BAI, Beck Anxiety Inventory; BDI, Beck Depression Inventory; BFI, Big Five Inventory; CES-D, Center for Epidemiologic Studies Depression Scale; GA, general anaesthesia; GAD, generalised anxiety disorder; GDS, Geriatric Depression Scale; HADS-A, Hospital Anxiety and Depression Scale–Anxiety; HAS, Hamilton Anxiety Scale; HRSD, Hamilton Rating Scale for Depression; IQR, interquartile range; NB, nerve block; NI, Need for Information Scale; NR, not reported; PHQ, Patient Health Questionnaire; PSS-10, Perceived Stress Scale—10-item version; PTSS, post-traumatic stress symptom; RA, regional anaesthesia; STAI, State-Trait Anxiety Inventory; TAC-24E, Tri-Axial Coping Scale 24-item; TIPI-J, 10-Item Personality Inventory—Japanese version.

Risk of bias	Authors and publication year	*n*	Country	Study design	Psychological instrument(s) used	Type of anaesthesia	Type of surgery	Age, mean (sd) or median (IQR) (yr)	Female patients (%)	Education background (%)
9	Liu and colleagues (2023)^45^	120	China	Prospective cohort	BAI	GA	Abdominal cancer	69 (66–73)	27.5%	NR
9	Ren and colleagues (2021)^32^	264	China	Prospective cohort	HADS-A	GA NB	Orthopaedic	74.2 (7.3)	71%	Illiteracy, 33.1% Primary or junior high school, 31.9% High school, college, or higher, 35%
9	Wang and colleagues (2025)^41^	156	China	Prospective cohort	GAD PHQ	GA	Urological	NR	0%	NR
8	Leung and colleagues (2023)^43^	180	USA	Prospective cohort	HADS-A GDS	GA RA	Mixed	72.25 (5.45)	49%	NR
8	Ackenbom and colleagues (2023)^44^	183	USA	Prospective cohort	BAI GDS	GA Local i.v.+sedation RA	Gynaecological	72.5 (6.1)	100%	Education, median 13 yr (12–16)
8	Fukunaga and colleagues (2022)^37^	168	Japan	Prospective cohort	STAI trait+state GDS TIPI-J TAC-24E	NR	Mixed	74.9 (6.1)	45%	NR
8	de Mul and colleagues (2022)^46^	255	the Netherlands, Switzerland, Belgium	Prospective cohort	HADS-A GDS PTSS	NR	Mixed	70 (68–74)	31%	NR
8	Chan and colleagues (2021)^39^	199	USA, Sweden, UK, Morocco	Prospective cohort	GDS	RA	Orthopaedic	81.9 (7.7)	72.9%	Less than high school. 38.2% High school. 38.2% Some college. 14.1% College or higher. education 9.5%
8	Janssen and colleagues (2021)^56^	265	the Netherlands, Belgium	Prospective cohort	CES-D	NR	Colorectal cancer+aortic	76 (73–81)	35%	NR
8	Rao and colleagues (2020)^47^	187	USA	Prospective cohort	GDS	NR	Cardiac+intervention	81.3 (6.4)	48.1%	NR
8	Tao and colleagues (2019)^28^	507	China	Prospective cohort	GDS	NR	Orthopaedic	77.2 (7.6)	65%	NR
8	Yamamoto and colleagues (2016)^31^	91	Japan	Prospective cohort	GDS	NR	Oesophageal cancer	78.4 (2.8)	17.6%	NR
8	Cheong and colleagues (2021)^38^	447	Malaysia	Prospective cohort	GDS	NR	Mixed	73.23 (5.94)	53.7%	≤6 yr of education, 43.2%
8	Baek and colleagues (2023)^48^	91	Republic of Korea	Prospective cohort	GDS	NR	Orthopaedic	76 (73–78)	72.2%	NR
8	Radinovic and colleagues (2014)^57^	277	Serbia	Prospective cohort	GDS	GA RA	Orthopaedic	78 (8.2)	74.4%	No formal education, 21.7% Elementary school, 22% Secondary school, 32.9% Post-secondary education, 23.5%
8	Itami and colleagues (2024)^42^	255	Japan	Prospective cohort	GDS	NR	Abdominal cancer	79	27.1%	NR
Low risk	Olofsson and colleagues (2018)^49^	135	Sweden	RCT	GDS	NR	Orthopaedic	83.1 (6.1)	74%	NR
Low risk	Milisen and colleagues (2020)^34^	190	Belgium	RCT	APAIS-A, APAIS-NFA GDS	GA	Cardiac	75.7 (5.9)	47.9%	<15 education years, 41.3% 16–18 education years, 40.7% College/university, 18%
Low risk	Umoh and colleagues (2025)^55^	157	USA	RCT	GDS	RA	Orthopaedic	84 [76,88]	73.8%	Elementary, 18% High school, 43% College, 26%
7	Ackenbom and colleagues (2021)^50^	72	USA	Prospective cohort	BAI GDS	GA	Gynaecological	72 (69–77)	100%	≤12 yr education, 34.7% >12–≤16 yr, 52.8% >16 yr, 12.5%
7	Banjongrewadee and colleagues (2020)^40^	429	Thailand	Prospective cohort	GDS NI PSS-10	GA RA Peripheral NB	Unspecified noncardiac	69.93 (6.87)	58.97%	Mean (sd), 6.43 (5.02) yr
7	Dogrul and colleagues (2020)^51^	108	Turkey	Prospective cohort	GDS	NR	Mixed	71	63%	NR
7	Khan and colleagues (2019)^36^	234	Canada	Prospective cohort	PHQ	GA	Cardiac	82.2 (6.7)	41%	NR
7	Koskderelioglu and colleagues (2017)^35^	109	Turkey	Prospective cohort	BDI	GA RA	Orthopaedic	77.5 (7.7)	39%	NR
7	Shin and colleagues (2016)^29^	78	Republic of Korea	Prospective cohort	HAS HRSD BFI	RA	Orthopaedic	81.6 (6.6)	80.8%	Mean (sd), 8.2 (4.6) yr
7	Mokutani and colleagues (2016)^33^	156	Japan	Retrospective cohort	GDS	NR	Colorectal cancer	80.2 (4.1)	43%	NR
7	Maekawa and colleagues (2016)^30^	517	Japan	Prospective cohort	GDS	RA Peripheral NB	Gastrointestinal cancer	79.3 (3.6)	32.1%	NR
7	Liang and colleagues (2014)^52^	232	Taiwan	Prospective cohort	GDS	NR	Orthopaedic	74.7 (7.8)	51.2%	Mean (sd) education, 5.8 (4.7) yr
7	Tai and colleagues (2015)^53^	485	China	Prospective cohort	GDS	RA	Urological	71.25 (2.35)	0%	NR
7	Deiner and colleagues (2021)^54^	167	USA	Prospective cohort	HADS	GA	Mixed	70 (67–74)	55.1%	Median education, 16 yr

Global cognition was most commonly assessed before surgery with cognitive screening tools such as Mini-Mental State Examination (MMSE)^28^, ^29^, ^30^, ^31^, ^32^, ^33^, ^34^, ^35^^,^^37^^,^^39^^,^^42^^,^^46^^,^^47^^,^^49^^,^^51^, ^52^, ^53^^,^^55^^,^^56^ or Montreal Cognitive Assessment (MoCA).^38^^,^^40^^,^^48^ Most studies assessed delirium after surgery with the Confusion Assessment Method (CAM),^28^, ^29^, ^30^, ^31^, ^32^, ^33^, ^34^, ^35^^,^^38^, ^39^, ^40^, ^41^, ^42^, ^43^^,^^45^, ^46^, ^47^, ^48^^,^^50^^,^^52^, ^53^, ^54^, ^55^^,^^57^ and timing of assessment was heterogeneous, ranging from not reported to reported every postoperative day until discharge. Two studies assessed dNCR/p-NCD with a neuropsychological test battery at 2 weeks or 3 months after surgery^50^^,^^54^ (Supplementary Table 2). Psychological factors were also assessed before surgery using standardised instruments such as the Geriatric Depression Scale (GDS) (Table 1). These variables were reported either as binary outcomes such as the presence of stress *vs* absence of stress, or as continuous scores, with varying cut-off scores.

### Participant characteristics

The reported mean or median age for all participants ranged from 69 to 84 yr, with a larger proportion of female patients. Across all studies, there were 1388 patients diagnosed with POD, and 45 patients with dNCR/p-NCD. Educational background was reported in 11 studies,^29^^,^^32^^,^^34^^,^^38^, ^39^, ^40^^,^^44^^,^^50^^,^^52^^,^^54^^,^^57^ ranging from illiteracy to university-level education (Table 1). Two studies included information on occupational background, citing roles as farmers and government employees.^40^^,^^45^

The surgical populations varied: most underwent orthopaedic surgery,^28^^,^^29^^,^^32^^,^^35^^,^^39^^,^^48^^,^^49^^,^^52^^,^^57^ followed by cancer surgery,^30^^,^^31^^,^^33^^,^^45^^,^^56^ mixed types of surgeries,^38^^,^^43^^,^^46^^,^^51^^,^^54^ cardiac surgery,^34^^,^^36^^,^^37^^,^^47^ gynaecological surgery,^44^^,^^50^ unspecified noncardiac surgery,^40^ and urological surgery,^53^ Types of anaesthesia included general anaesthesia, regional anaesthesia, or a combination with peripheral nerve block.

### Association between psychological factors and postoperative cognitive outcomes

Four categories of psychological factors were identified: depression,^28^, ^29^, ^30^, ^31^^,^^33^, ^34^, ^35^, ^36^, ^37^, ^38^, ^39^, ^40^, ^41^, ^42^, ^43^, ^44^^,^^46^, ^47^, ^48^, ^49^, ^50^, ^51^, ^52^, ^53^, ^54^, ^55^, ^56^, ^57^ anxiety,^29^^,^^32^^,^^34^^,^^37^^,^^41^^,^^43^, ^44^, ^45^, ^46^^,^^50^^,^^54^ personality traits such as neuroticism,^29^^,^^37^^,^^40^ and stress-related factors.^37^^,^^40^^,^^46^ The latter included both stress symptoms and coping style. POD was the most reported outcome, present in all 30 studies. Incidence ranged from 9% to 55.6% (Supplementary Table 2), with the highest rates reported in two studies focusing on patients with hip fractures (>50%).^29^^,^^49^ dNCR and p-NCD were reported in one study each,^50^^,^^54^ both referring to the outcome as POCD (Supplementary Table 3).

There was evidence for an association between depression, anxiety, neuroticism, and stress symptoms on postoperative cognitive outcomes (*P*<0.001, 28 studies). The albatross plot is shown in Figure 4. The effect contours represent approximate standardised mean differences and should not be interpreted as pooled effect estimates.

#### Depression

In studies reporting multivariate adjustments, depressive symptoms showed no statistically significant association with POD (OR, 1.08; 95% CI, 0.97–1.2) (Figs 2 and 3). When compiling *P*-values, a positive direction of effect between depression and POD and dNCR respectively was observed.^28^^,^^33^^,^^35^^,^^39^^,^^43^^,^^44^^,^^50^^,^^51^^,^^53^

#### Anxiety

Anxiety showed no statistically significant association with POD (OR, 0.99; 95% CI, 0.94–1.04) (Figs 2 and 3). When compiling *P*-values, a positive association was observed for anxiety and POD in three studies^32^^,^^44^^,^^45^ and a negative association in one.^43^

#### Personality traits

Personality traits showed no statistically significant association with POD (OR, 0.94; 95% CI, 0.54–1.65) (Figs 2 and 3). In *P*-value synthesis, agreeableness and conscientiousness^29^^,^^37^ showed a negative effect, whereas neuroticism showed a positive direction of effect.^29^

#### Stress-related factors

One study showed a positive association between the presence of stress symptoms and POD.^40^ Vote counting based on effect direction identified one negative association with stress symptoms,^46^ and one with coping behaviours, including avoidance-like thinking with POD.^37^

### Publication bias

For the multivariate meta-analysis, Egger’s test for funnel plot asymmetry generated a *t*-value of 0.9418 (*df*=20, *P*=0.3575). The limit estimate (as the standard error approaches zero) was β=−0.0246 (95% CI, −0.1145 to 0.0654). For the univariate meta-analysis, the *t*-value was 4.97 (*df*=2, *P*=0.0383) with a limit estimate of β=0.9808 (95% CI, 0.9034–1.0583). The funnel plot is presented in Supplementary Figure 2.

## Discussion

This systematic review synthesised findings from 30 studies investigating the association between psychological factors and postoperative cognitive outcomes in more than 6700 older adults undergoing various types of surgery, ranging from orthopaedic to cancer surgeries. Variation in cultural context and healthcare settings may have influenced how psychological constructs were operationalised, measured, and interpreted across studies.

Depression was the most frequently reported psychological risk factor of POD, and the estimates were lower than those reported in a previous meta-analysis,^15^ likely reflecting the use of multivariable-adjusted effect sizes. Despite this, depression may remain clinically relevant to detect, particularly given the high baseline risk of POD in older adults and its established links to adverse outcomes, including prolonged length of stay, mortality, institutionalisation and post-traumatic stress disorder.^58^^,^^59^

A growing body of evidence suggests that age-related immunological changes (inflammaging), late-life depression, and delirium are each associated with inflammatory processes,^60^, ^61^, ^62^ raising the possibility that they may share common underlying pathways. Persistent low-grade inflammation, indexed by repeated elevations in C-reactive protein (CRP), has been associated with depressive symptoms in older adults, particularly women.^61^ Additionally, measurement bias may contribute to missed detection of depression. For example, the Patient Health Questionnaire (PHQ-9), a commonly used screening tool, has a standard cut-off of 10 that may not be optimal in older adults. Evidence suggests that lowering this threshold improves sensitivity and diagnostic accuracy in older adults,^63^ and studies using the conventional cut-off may underestimate the impact of depression.

Anxiety was not significantly associated with POD in the meta-analysis, whereas the *P*-value synthesis showed mixed evidence with both positive and negative associations. These findings diverge from earlier studies reporting increased odds of POD in mixed-aged patients with preoperative anxiety,^16^ and of p-NCD after cancer surgery.^10^ This may partly be attributed to variability in how anxiety was measured. Common instruments such as the State-Trait Anxiety Inventory (STAI) and Hospital Anxiety and Depression Scale (HADS) were developed in younger populations, and both may lack validity in older adults. Instruments such as the Rating Anxiety in Dementia (RAID) or Geriatric Anxiety Inventory (GAI) for cognitively intact older adults could offer more age-appropriate alternatives.^64^

Furthermore, older adults tend to report higher emotional well-being and greater psychological resiliency than younger adults, even in the context of severe illness.^65^ However, state anxiety in the preoperative setting is a modifiable risk factor that may be exacerbated by poor communication, procedural uncertainty, and mistrust.^66^ These aspects are amenable to educational and psychological interventions.^67^

Fewer studies assessed personality traits and stress-related factors, and findings were heterogeneous. Stable personality traits may influence postoperative cognitive outcomes, but this remains an underdeveloped area of research. Higher neuroticism was linked to a positive effect of POD, whereas agreeableness and conscientiousness were linked to a negative effect. These results align with evidence from a systematic review linking neuroticism to increased risk of mild cognitive impairment and dementia, whereas conscientiousness appears to be a protective factor.^68^ Possible mechanisms could be elevated cortisol responses, altered neurotransmitter activity, and increased levels of pro-inflammatory markers.^69^ Moreover, a recent population-based cohort study found that the presence of a personality disorder in late life was associated with a greater risk (risk ratio 25) of receiving a diagnosis of delirium, or dementia.^70^

Incidence of POD was the most reported postoperative cognitive outcome. Most included studies used validated instruments to assess delirium as outlined by the European Society of Anaesthesiology guidelines.^71^ However, as the timing of assessment was highly heterogeneous, in some instances, it may have led to an underdiagnosis of POD.

To the best of our knowledge, this is the first systematic review to compile and synthesise evidence on the association between multiple psychological factors and POD, dNCR, and p-NCD. In addition, all studies were critically appraised, and only those assessed as having low risk of bias were included, strengthening the credibility of our evidence. Although this may limit generalisability to some extent, our review includes diverse surgical populations and excluded a small number of studies for moderate or high risk of bias. Overall, the resulting evidence base can be regarded as robust and broadly applicable. In accordance with the Cochrane Handbook^24^ and SWiM guidelines,^20^ we applied structured alternative synthesis methods, rather than relying on methodologically weaker narrative summaries. Our approach mitigates a common limitation in systematic reviews, in which studies not included in the meta-analysis are often excluded or insufficiently reported.^20^^,^^72^

### Limitations

Several limitations of the primary studies warrant caution. Most studies were observational in design, consistent with the types of research questions being addressed; thus, confounding factors will remain a limitation in the findings. Most studies did not report cognitive reserve variables including educational or occupational background in the included population, despite their known influence on cognitive decline in older adults.^73^^,^^74^ Reporting of outcomes was in some studies incomplete, and the timing of assessment of POD varied widely. The scarcity of studies on dNCR and p-NCD is a major gap, with all studies primarily focusing on POD.

Limitations within the review should be acknowledged. To ensure the inclusion of all eligible studies in our synthesis, we compiled *P*-values and vote counting based on direction of effect. However, these synthesis methods have limitations, as they do not provide information on the magnitude of effects.^24^ We also restricted inclusion to studies published in English, which may have introduced language bias.

### Future directions

Future studies should use validated instruments tailored to older adults and adopt the newer definitions of cognitive outcomes, with standardised diagnostic criteria for dNCR and p-NCD that enable better cross-study comparability. Prospective longitudinal cohort designs are well suited to capture the trajectory of postoperative cognitive outcomes in older adults. Given the fluctuating nature of delirium, assessing POD with validated instruments on each postoperative day and each clinical shift is needed. Studies investigating postoperative cognitive outcomes should incorporate depression screening, delirium screening, in combination with performance-based cognitive tests, Instrumental Activities Daily Living evaluation, and subjective reports of cognitive change from patients or informants. For studies with frail older individuals, a suitable multidisciplinary approach would be following the Comprehensive Geriatric Assessment (CGA).^62^ The extent to which pharmacological treatment or cognitive behavioural therapy (CBT) can reduce POD risk is unclear, warranting further investigation into their potential risk-modifying effects. Notably, CBT in patients undergoing cardiac surgery has been shown to improve health-related quality of life and heart rate variability,^75^ suggesting benefits for postoperative outcomes.

From a statistical perspective, future studies should pre-define covariates based on clinical relevance rather than selecting them *post hoc* based on statistical significance.^76^ Variables such as global cognition and educational background, which are well-established predictors of cognitive performance in older adults,^77^^,^^78^ should be included in multivariable models irrespective of *P*-values. This approach could reduce the risk of model overfitting and improves the robustness and reproducibility of findings.

### Conclusions

This systematic review and meta-analysis highlight that preoperative depression is the most consistently reported psychological risk factor for POD in older adults, with potential clinical relevance despite non-significant statistical associations. Evidence for anxiety, personality traits, and stress-related factors remains mixed or limited, reflecting heterogeneity in measurement and study design. We emphasise the need for age-appropriate assessment tools, standardised diagnostic criteria, and longitudinal designs to better capture postoperative cognitive outcomes. Although the current evidence base seem robust and broadly applicable, future research should integrate multidimensional cognitive assessments, consider confounding factors such as cognitive reserve, and explore targeted interventions, including pharmacological or psychological treatment, to mitigate risk and improve cognitive outcomes after surgery.

## Authors’ contributions

Conceptualisation: AA, LB, UN

Methodology: AA, AF, LB, UN

Search strategy: AA, LB, UN

Literature search: AA, AF, LB, UN

Formal analysis, data curation: AA, AF

Writing, original draft: AA

Writing, review and editing: all authors

Supervision: AF, GM, JE, LB, UN

## Funding

Strategic Research Area Health Care Science (SFO-V) (INT-2024-0002 and 2-3226/2023); Karolinska Institutet (Research School in Health Science; 2020-02641).

## Declaration of interest

The authors declare that they have no conflicts of interest.
