# Supplementary material for: Psychological factors associated with postoperative cognitive outcomes in older adults: a systematic review and meta-analysis
Source: Br J Anaesth. 2026 Feb 27;136(5):1482–94. doi: 10.1016/j.bja.2026.01.031 (PMC13197908; doi:10.1016/j.bja.2026.01.031)
Supplement: Supplementary file 1 [file mmc1.docx]

**Supplementary Table S1.** Full search strategies for all databases

1. Medline

| Interface: **Ovid MEDLINE(R) ALL** content coverage from 1946  Date of Search: 1 Oktober 2024  Number of hits: 3,751  Comment: In Ovid, two or more words are automatically searched as phrases; i.e. no quotation marks are needed | Field labels   - exp/ = exploded MeSH term - / = non exploded MeSH term - .ti,ab,kf. = title, abstract and author keywords - adjx = within x words, regardless of order - * = truncation of word for alternate endings - ? = 0-1 letter/number - # = 1 letter/number |
| --- | --- |
| Database(s): **Ovid MEDLINE(R) ALL**1946 to September 30, 2024 Search Strategy:   \| **#** \| **Searches** \| **Results** \| \| --- \| --- \| --- \| \| 1 \| Depression/ \| 162103 \| \| 2 \| exp Depressive Disorder/ \| 126161 \| \| 3 \| Anxiety/ \| 115738 \| \| 4 \| exp Anxiety Disorders/ \| 94724 \| \| 5 \| Mental Health/ \| 69291 \| \| 6 \| exp "Quality of Life"/ \| 294676 \| \| 7 \| Personal Satisfaction/ \| 25761 \| \| 8 \| Stress Disorders, Post-Traumatic/ \| 43973 \| \| 9 \| Personality/ \| 43002 \| \| 10 \| Self Efficacy/ \| 25723 \| \| 11 \| Catastrophization/ \| 2065 \| \| 12 \| Pain Perception/ \| 3375 \| \| 13 \| Coping Skills/ \| 540 \| \| 14 \| Stress, Psychological/ \| 137841 \| \| 15 \| Psychological Distress/ \| 5117 \| \| 16 \| Resilience, Psychological/ \| 10200 \| \| 17 \| Adaptation, Psychological/ \| 106853 \| \| 18 \| Fear/ \| 40536 \| \| 19 \| ((psycholog* or psychiatric*) adj3 (adaptation* or condition* or distress or factor* or health* or ill being or illness* or symptom* or status or stress or wellbeing or well-being or wellness)).ti,ab,kf. \| 176829 \| \| 20 \| ((emotion* or mental) adj3 (distress or health* or ill being or state* or symptom* or stress or wellbeing or well-being or wellness)).ti,ab,kf. \| 362266 \| \| 21 \| (coping adj3 (anticipatory or approach* or behavio* or cognitiv* or constructive or primary or proactive or skill* or social or strateg*)).ti,ab,kf. \| 34044 \| \| 22 \| (pain adj3 (catastrophi* or perception* or rumination*)).ti,ab,kf. \| 16286 \| \| 23 \| (antidepress* or anxiet* or anxious* or depression* or depressed or depressive or fear or "locus of control" or mental* outcome* or personality or personal satisfaction or PTSD or post-traumatic stress or resilien* or self-efficacy or stress immunit* or worry*).ti,ab,kf. \| 1043328 \| \| 24 \| (expectation* adj3 recover*).ti,ab,kf. \| 708 \| \| 25 \| (life adj2 satisf*).ti,ab,kf. \| 16875 \| \| 26 \| ((preanesthe* or pre-anesthe* or preanaesthe* or pre-anaesthe* or preoperative* or pre-operative* or presurg* or pre-surg*) adj3 cogniti*).ti,ab,kf. \| 730 \| \| 27 \| or/1-26 \| 1831010 \| \| 28 \| Psychiatric Status Rating Scales/ \| 80460 \| \| 29 \| "Mental Status and Dementia Tests"/ \| 3346 \| \| 30 \| Geriatric Assessment/ \| 33823 \| \| 31 \| Risk Assessment/ \| 318006 \| \| 32 \| exp Psychological Tests/ \| 354848 \| \| 33 \| "Predictive Value of Tests"/ \| 228499 \| \| 34 \| Mental Status Schedule/ \| 6290 \| \| 35 \| Psychometrics/ \| 92862 \| \| 36 \| Logistic Models/ \| 154254 \| \| 37 \| ((anxiet* or anxious* or cogni* or depress* or geriatric* or health* or mental* or neurocogni* or neuropsych* or psych* or personality) adj10 (assess* or evaluat* or instrument* or measure* or questionnaire* or psychometric* or scale* or score* or screen* or survey* or test*)).ti,ab,kf. \| 1468677 \| \| 38 \| (predict* adj2 (value* or variable*)).ti,ab,kf. \| 209361 \| \| 39 \| (logistic adj2 (model* or regression*)).ti,ab,kf. \| 476521 \| \| 40 \| or/28-39 \| 2635429 \| \| 41 \| Postoperative Cognitive Complications/ \| 622 \| \| 42 \| Emergence Delirium/ \| 898 \| \| 43 \| (agitated emergence or emergence agitation* or emergence delirium or emergence excitement* or postanesthe* excitement* or post-anesthe* excitement* or postanaesthe* excitement* or post-anaesthe* excitement*).ti,ab,kf. \| 1125 \| \| 44 \| ((postoperative* or post-operative*) adj3 confusion*).ti,ab,kf. \| 239 \| \| 45 \| or/41-44 \| 2535 \| \| 46 \| Postoperative Period/ \| 57305 \| \| 47 \| Postoperative Care/ \| 61064 \| \| 48 \| Postanesthesia Nursing/ \| 1100 \| \| 49 \| Postoperative Complications/ \| 414146 \| \| 50 \| (postanesthe* or postanaesthe* or postoperative* or postsurger*).ti,ab,kf. \| 721903 \| \| 51 \| ((after* or follow* or post) adj4 (anesthe* or anaesthe* or operat* or surger* or surgical or procedure*)).ti,ab,kf. \| 841008 \| \| 52 \| or/46-51 \| 1558754 \| \| 53 \| Cognition/ \| 136796 \| \| 54 \| Cognitive Dysfunction/ \| 42679 \| \| 55 \| Cognition Disorders/ or Neurocognitive Disorders/ \| 76768 \| \| 56 \| Delirium/ \| 13048 \| \| 57 \| "Recovery of Function"/ \| 61011 \| \| 58 \| ((postoperative complication* or post-operative complication*) and (cogniti* or neurocogniti* or neuropsycho* or neuro-psycho*)).ti,ab,kf. \| 745 \| \| 59 \| ((cogniti* or neurocogniti* or neuropsycho* or neuro-psycho*) adj3 (complication* or change* or condition* or decline or deficit* or disorder* or dysfunction* or failure* or function* or impairment* or outcome* or recover* or score* or state* or status* or symptom*)).ti,ab,kf. \| 310416 \| \| 60 \| (delirium or POCD).ti,ab,kf. \| 24428 \| \| 61 \| or/53-60 \| 496772 \| \| 62 \| 52 and 61 \| 35670 \| \| 63 \| 45 or 62 \| 36503 \| \| 64 \| 27 and 40 and 63 \| 4226 \| \| 65 \| (exp Child/ or exp Infant/ or exp Adolescent/ or (child? or children* or childhood or adolescen* or infant* or neonat* or juvenile* or p?ediatric*).ti.) not (exp Aged/ or Middle Aged/ or (aged or aging or ageing or female* or male* or middle age* or geriatric* or old or older* or elder* or men* or man or senior* or wom?n*).ti.) \| 3091251 \| \| 66 \| 64 not 65 \| 3894 \| \| 67 \| 66 not (animals not humans).sh. \| 3751 \| | |

2. Embase

| Interface: **embase.com** content coverage from 1947  Date of Search: 1 Oktober 2024  Number of hits: 4,547  Comment: Emtree is the controlled vocabulary in Embase | Field labels   - /exp = exploded Emtree term - /de = non exploded Emtree term - ti,ab,kw = title, abstract and author keywords - NEAR/x = within x words, regardless of order - * = truncation of word for alternate endings - # = 0-1 letter/number - ? = 1 letter/number |
| --- | --- |
| \| **No.** \| **Query** \| **Results** \| \| --- \| --- \| --- \| \| #71 \| #70 AND ('Article'/it OR 'Article in Press'/it OR 'Conference Paper'/it OR 'Erratum'/it OR 'Preprint'/it OR 'Review'/it OR 'Short Survey'/it) \| 4547 \| \| #70 \| #69 NOT ([animals]/lim NOT [humans]/lim) \| 6207 \| \| #69 \| #61 NOT #68 \| 6479 \| \| #68 \| #64 NOT #67 \| 3995156 \| \| #67 \| #65 OR #66 \| 6949536 \| \| #66 \| aged:ti OR aging:ti OR ageing:ti OR female*:ti OR male*:ti OR 'middle age*':ti OR geriatric*:ti OR old:ti OR older*:ti OR elder*:ti OR men*:ti OR man:ti OR senior*:ti OR wom$n*:ti \| 2044620 \| \| #65 \| 'middle aged'/de OR 'aged'/exp \| 5546840 \| \| #64 \| #62 OR #63 \| 4959500 \| \| #63 \| child$:ti OR children*:ti OR childhood:ti OR adolescen*:ti OR infant*:ti OR neonat*:ti OR juvenile*:ti OR p$ediatric*:ti \| 2117886 \| \| #62 \| 'juvenile'/exp \| 4603491 \| \| #61 \| #26 AND #38 AND #60 \| 7081 \| \| #60 \| #43 OR #59 \| 49971 \| \| #59 \| #49 AND #58 \| 47479 \| \| #58 \| #50 OR #51 OR #52 OR #53 OR #54 OR #55 OR #56 OR #57 \| 788375 \| \| #57 \| delirium:ti,ab,kw OR pocd:ti,ab,kw \| 37456 \| \| #56 \| ((cogniti* OR neurocogniti* OR neuropsycho* OR 'neuro psycho*') NEAR/3 (complication* OR change* OR condition* OR decline OR deficit* OR disorder* OR dysfunction* OR failure* OR function* OR impairment* OR outcome* OR recover* OR score* OR state* OR status* OR symptom*)):ti,ab,kw \| 446057 \| \| #55 \| ('postoperative complication*':ti,ab,kw OR 'post-operative complication*':ti,ab,kw) AND (cogniti*:ti,ab,kw OR neurocogniti*:ti,ab,kw OR neuropsycho*:ti,ab,kw OR 'neuro psycho*':ti,ab,kw) \| 1184 \| \| #54 \| 'convalescence'/de \| 60850 \| \| #53 \| 'delirium'/de \| 39137 \| \| #52 \| 'disorders of higher cerebral function'/de \| 4190 \| \| #51 \| 'cognitive defect'/de \| 237308 \| \| #50 \| 'cognition'/de \| 334541 \| \| #49 \| #44 OR #45 OR #46 OR #47 OR #48 \| 2177356 \| \| #48 \| ((after* OR follow* OR post) NEAR/4 (anesthe* OR anaesthe* OR operat* OR surger* OR surgical OR procedure*)):ti,ab,kw \| 1255994 \| \| #47 \| postanesthe*:ti,ab,kw OR postanaesthe*:ti,ab,kw OR postoperative*:ti,ab,kw OR postsurger*:ti,ab,kw \| 1111492 \| \| #46 \| 'postoperative complication'/de \| 468342 \| \| #45 \| 'postanesthesia nursing'/de \| 953 \| \| #44 \| 'postoperative period'/de OR 'postanesthesia care'/de OR 'postoperative care'/de \| 404473 \| \| #43 \| #39 OR #40 OR #41 OR #42 \| 8637 \| \| #42 \| ((postoperative* OR 'post operative*') NEAR/3 confusion*):ti,ab,kw \| 327 \| \| #41 \| 'agitated emergence':ti,ab,kw OR 'emergence agitation*':ti,ab,kw OR 'emergence delirium':ti,ab,kw OR 'emergence excitement*':ti,ab,kw OR 'postanesthe* excitement*':ti,ab,kw OR 'post-anesthe* excitement*':ti,ab,kw OR 'postanaesthe* excitement*':ti,ab,kw OR 'post-anaesthe* excitement*':ti,ab,kw \| 1441 \| \| #40 \| 'emergence agitation'/de OR 'postoperative delirium'/de \| 5806 \| \| #39 \| 'postoperative cognitive dysfunction'/de \| 2328 \| \| #38 \| #27 OR #28 OR #29 OR #30 OR #31 OR #32 OR #33 OR #34 OR #35 OR #36 OR #37 \| 3813985 \| \| #37 \| (logistic NEAR/2 (model* OR regression*)):ti,ab,kw \| 678515 \| \| #36 \| (predict* NEAR/2 (value* OR variable*)):ti,ab,kw \| 304730 \| \| #35 \| ((anxiet* OR anxious* OR cogni* OR depress* OR geriatric* OR health* OR mental* OR neurocogni* OR neuropsych* OR psych* OR personality) NEAR/10 (assess* OR evaluat* OR instrument* OR measure* OR questionnaire* OR psychometric* OR scale* OR score* OR screen* OR survey* OR test*)):ti,ab,kw \| 1969119 \| \| #34 \| 'statistical model'/de \| 177725 \| \| #33 \| 'psychometry'/exp \| 121011 \| \| #32 \| 'predictive value'/de \| 274344 \| \| #31 \| 'psychologic test'/exp \| 248679 \| \| #30 \| 'risk assessment'/de \| 772132 \| \| #29 \| 'geriatric assessment'/de \| 22415 \| \| #28 \| 'dementia assessment'/exp \| 65591 \| \| #27 \| 'psychological rating scale'/de \| 22923 \| \| #26 \| #1 OR #2 OR #3 OR #4 OR #5 OR #6 OR #7 OR #8 OR #9 OR #10 OR #11 OR #12 OR #13 OR #14 OR #15 OR #16 OR #17 OR #18 OR #19 OR #20 OR #21 OR #22 OR #23 OR #24 OR #25 \| 2996573 \| \| #25 \| ((preanesthe* OR 'pre anesthe*' OR preanaesthe* OR 'pre anaesthe*' OR preoperative* OR 'pre operative*' OR presurg* OR 'pre surg*') NEAR/3 cogniti*):ti,ab,kw \| 1019 \| \| #24 \| (life NEAR/2 satisf*):ti,ab,kw \| 19745 \| \| #23 \| (expectation* NEAR/3 recover*):ti,ab,kw \| 963 \| \| #22 \| antidepress*:ti,ab,kw OR anxiet*:ti,ab,kw OR anxious*:ti,ab,kw OR depression*:ti,ab,kw OR depressed:ti,ab,kw OR depressive:ti,ab,kw OR fear:ti,ab,kw OR 'locus of control':ti,ab,kw OR 'mental* outcome*':ti,ab,kw OR personality:ti,ab,kw OR 'personal satisfaction':ti,ab,kw OR ptsd:ti,ab,kw OR 'post-traumatic stress':ti,ab,kw OR resilien*:ti,ab,kw OR 'self efficacy':ti,ab,kw OR 'stress immunit*':ti,ab,kw OR worry*:ti,ab,kw \| 1377392 \| \| #21 \| (pain NEAR/3 (catastrophi* OR perception* OR rumination*)):ti,ab,kw \| 22383 \| \| #20 \| (coping NEAR/3 (anticipatory OR approach* OR behavio* OR cognitiv* OR constructive OR primary OR proactive OR skill* OR social OR strateg*)):ti,ab,kw \| 43071 \| \| #19 \| ((emotion* OR mental) NEAR/3 (distress OR health* OR 'ill being' OR state* OR symptom* OR stress OR wellbeing OR 'well being' OR wellness)):ti,ab,kw \| 455730 \| \| #18 \| ((psycholog* OR psychiatric*) NEAR/3 (adaptation* OR condition* OR distress OR factor* OR health* OR 'ill being' OR illness* OR symptom* OR status OR stress OR wellbeing OR 'well being' OR wellness)):ti,ab,kw \| 228613 \| \| #17 \| 'fear'/de \| 87244 \| \| #16 \| 'psychological adjustment'/de \| 4077 \| \| #15 \| 'psychological resilience'/exp \| 13441 \| \| #14 \| 'mental stress'/exp \| 223542 \| \| #13 \| 'coping'/exp \| 90045 \| \| #12 \| 'nociception'/de \| 51624 \| \| #11 \| 'catastrophizing'/de \| 5599 \| \| #10 \| 'self concept'/de \| 118085 \| \| #9 \| 'personality'/de \| 72162 \| \| #8 \| 'posttraumatic stress disorder'/de \| 88654 \| \| #7 \| 'satisfaction'/de OR 'life satisfaction'/de \| 96613 \| \| #6 \| 'wellbeing'/de OR 'emotional well-being'/de \| 95534 \| \| #5 \| 'quality of life'/exp \| 715656 \| \| #4 \| 'mental health'/exp \| 272601 \| \| #3 \| 'anxiety disorder'/exp \| 353761 \| \| #2 \| 'anxiety'/de \| 321993 \| \| #1 \| 'depression'/exp \| 701025 \| | |

3. Web of Science Core Collection

| Interface: **Clarivate Analytics**  Editions and content coverage years= A&HCI - 1975 , ESCI -2019 , SCI-EXPANDED - 1945 , SSCI - 1945  Date of Search: 1 Oktober 2024  Number of hits: 2,942 | Field labels   - TS/Topic = title, abstract, author keywords and Keywords Plus - TI= title - AB = abstract - AK = author keywords - NEAR/x = within x words, regardless of order - * = truncation of word for alternate endings - # = 0-1 letter/number - ? = 1 letter/number   Note: the *Exact search*-function was used for all the searches |
| --- | --- |
| \| **#** \| **Search Query** \| **Results** \| \| --- \| --- \| --- \| \| 1 \| TS=((psycholog* OR psychiatric*) NEAR/3 (adaptation* OR condition* OR distress OR factor* OR health* OR "ill being" OR illness* OR symptom* OR status OR stress OR wellbeing OR well-being OR wellness)) \| 233212 \| \| 2 \| TS=((emotion* OR mental) NEAR/3 (distress OR health* OR "ill being" OR state* OR symptom* OR stress OR wellbeing OR well-being OR wellness)) \| 496413 \| \| 3 \| TS=(coping NEAR/3 (anticipatory OR approach* OR behavio* OR cognitiv* OR constructive OR primary OR proactive OR skill* OR social OR strateg*)) \| 52690 \| \| 4 \| TS=(pain NEAR/3 (catastrophi* OR perception* OR rumination*)) \| 17953 \| \| 5 \| TS=(antidepress* OR anxiet* OR anxious* OR depression* OR depressed OR depressive OR fear OR "locus of control" OR "mental* outcome*" OR personality OR "personal satisfaction" OR PTSD OR "post-traumatic stress" OR resilien* OR self-efficacy OR "stress immunit*" OR worry*) \| 1687778 \| \| 6 \| TS=(expectation* NEAR/3 recover*) \| 1045 \| \| 7 \| TS=(life NEAR/2 satisf*) \| 36689 \| \| 8 \| TS=((preanesthe* OR pre-anesthe* OR preanaesthe* OR pre-anaesthe* OR preoperative* OR pre-operative* OR presurg* OR pre-surg*) NEAR/3 cogniti*) \| 882 \| \| 9 \| #1 OR #2 OR #3 OR #4 OR #5 OR #6 OR #7 OR #8 \| 2120546 \| \| 10 \| TS=((anxiet* OR anxious* OR cogni* OR depress* OR geriatric* OR health* OR mental* OR neurocogni* OR neuropsych* OR psych* OR personality) NEAR/10 (assess* OR evaluat* OR instrument* OR measure* OR questionnaire* OR psychometric* OR scale* OR score* OR screen* OR survey* OR test*)) \| 1654034 \| \| 11 \| TS=(predict* NEAR/2 (value* OR variable*)) \| 293336 \| \| 12 \| TS=(logistic NEAR/2 (model* OR regression*)) \| 505345 \| \| 13 \| #12 OR #11 OR #10 \| 2311968 \| \| 14 \| TS=("agitated emergence" OR "emergence agitation*" OR "emergence delirium" OR "emergence excitement*" OR "postanesthe* excitement*" OR "post-anesthe* excitement*" OR "postanaesthe* excitement*" OR "post-anaesthe* excitement*") \| 1369 \| \| 15 \| TS=((postoperative* OR post-operative*) NEAR/3 confusion*) \| 266 \| \| 16 \| #14 OR #15 \| 1635 \| \| 17 \| TS=(postanesthe* OR postanaesthe* OR postoperative* OR postsurger*) \| 628760 \| \| 18 \| TS=((after* OR follow* OR post) NEAR/4 (anesthe* OR anaesthe* OR operat* OR surger* OR surgical OR procedure*)) \| 815136 \| \| 19 \| #17 OR #18 \| 1222853 \| \| 20 \| TS=(("postoperative complication*" OR "post-operative complication*") AND (cogniti* OR neurocogniti* OR neuropsycho* OR neuro-psycho*)) \| 830 \| \| 21 \| TS=((cogniti* OR neurocogniti* OR neuropsycho* OR neuro-psycho*) NEAR/3 (complication* OR change* OR condition* OR decline OR deficit* OR disorder* OR dysfunction* OR failure* OR function* OR impairment* OR outcome* OR recover* OR score* OR state* OR status* OR symptom*)) \| 403427 \| \| 22 \| TS=(delirium OR POCD) \| 29345 \| \| 23 \| #20 OR #21 OR #22 \| 425790 \| \| 24 \| #23 AND #19 \| 19560 \| \| 25 \| #24 OR #16 \| 20309 \| \| 26 \| #25 AND #13 AND #9 \| 3167 \| \| 27 \| TI=(child$ OR children* OR childhood OR adolescen* OR infant* OR neonat* OR juvenile* OR p$ediatric*) \| 2010780 \| \| 28 \| TI=(aged OR aging OR ageing OR female* OR male* OR "middle age*" OR geriatric* OR old OR older* OR elder* OR men* OR man OR senior* OR wom$n*) \| 2438415 \| \| 29 \| #27 NOT #28 \| 1891053 \| \| 30 \| #26 NOT #29 \| 2942 \| | |

4. PsycInfo

| Interface: **EBSCOhost** - content coverage from 1806  Date of Search: 1 Oktober 2024  Number of hits: 920 | Field labels   - DE = subject heading - TI = title - AB = abstract - KW = author keywords - Nx = within x words, regardless of order - * = truncation of word for alternate endings - ? = 0-1 letter/number - # = 1 letter/number   Note: sometimes “quotation marks” are needed for single search terms to avoid automatic term mapping (lemmatization) |
| --- | --- |
| \| **#** \| **Query** \| **Limiters/Expanders** \| **Results** \| \| --- \| --- \| --- \| --- \| \| S59 \| S57 NOT S58 \| Expanders - Apply equivalent subjects Search modes - Find all my search terms \| 920 \| \| S58 \| TI ( (child# OR children* OR childhood OR adolescen* OR infant* OR neonat* OR juvenile* OR p#ediatric*) ) NOT TI ( (aged OR aging OR ageing OR female* OR male* OR "middle age*" OR geriatric* OR old OR older* OR elder* OR men* OR man OR senior* OR wom#n*) ) \| Expanders - Apply equivalent subjects Search modes - Find all my search terms \| 533,150 \| \| S57 \| S23 AND S39 AND S56 \| Expanders - Apply equivalent subjects Search modes - Find all my search terms \| 969 \| \| S56 \| S42 OR S55 \| Expanders - Apply equivalent subjects Search modes - Find all my search terms \| 4,026 \| \| S55 \| S46 AND S54 \| Expanders - Apply equivalent subjects Search modes - Find all my search terms \| 3,990 \| \| S54 \| S47 OR S48 OR S49 OR S50 OR S51 OR S52 OR S53 \| Expanders - Apply equivalent subjects Search modes - Find all my search terms \| 283,844 \| \| S53 \| TI ( (delirium OR POCD) ) OR AB ( (delirium OR POCD) ) OR KW ( (delirium OR POCD) ) \| Expanders - Apply equivalent subjects Search modes - Find all my search terms \| 8,208 \| \| S52 \| TI ( ((cogniti* OR neurocogniti* OR neuropsycho* OR neuro-psycho*) N3 (complication* OR change* OR condition* OR decline OR deficit* OR disorder* OR dysfunction* OR failure* OR function* OR impairment* OR outcome* OR recover* OR score* OR state* OR status* OR symptom*)) ) OR AB ( ((cogniti* OR neurocogniti* OR neuropsycho* OR neuro-psycho*) N3 (complication* OR change* OR condition* OR decline OR deficit* OR disorder* OR dysfunction* OR failure* OR function* OR impairment* OR outcome* OR recover* OR score* OR state* OR status* OR symptom*)) ) OR KW ( ((cogniti* OR neurocogniti* OR neuropsycho* OR neuro-psycho*) N3 (complication* OR change* OR condition* OR decline OR deficit* OR disorder* OR dysfunction* OR failure* OR function* OR impairment* OR outcome* OR recover* OR score* OR state* OR status* OR symptom*)) ) \| Expanders - Apply equivalent subjects Search modes - Find all my search terms \| 205,992 \| \| S51 \| TI ( (("postoperative complication*" OR "post-operative complication*" ) AND (cogniti* OR neurocogniti* OR neuropsycho* OR neuro-psycho*)) ) OR AB ( (("postoperative complication*" OR "post-operative complication*" ) AND (cogniti* OR neurocogniti* OR neuropsycho* OR neuro-psycho*)) ) OR KW ( (("postoperative complication*" OR "post-operative complication*" ) AND (cogniti* OR neurocogniti* OR neuropsycho* OR neuro-psycho*)) ) \| Expanders - Apply equivalent subjects Search modes - Find all my search terms \| 78 \| \| S50 \| DE "Neurocognitive Disorders" \| Expanders - Apply equivalent subjects Search modes - Find all my search terms \| 5,625 \| \| S49 \| DE "Delirium" \| Expanders - Apply equivalent subjects Search modes - Find all my search terms \| 4,949 \| \| S48 \| DE "Cognitive Impairment" \| Expanders - Apply equivalent subjects Search modes - Find all my search terms \| 47,402 \| \| S47 \| (DE "Cognition" OR DE "Neurocognition") \| Expanders - Apply equivalent subjects Search modes - Find all my search terms \| 94,494 \| \| S46 \| S43 OR S44 OR S45 \| Expanders - Apply equivalent subjects Search modes - Find all my search terms \| 37,192 \| \| S45 \| TI ( ((after* OR follow* OR post) N4 (anesthe* OR anaesthe* OR operat* OR surger* OR surgical OR procedure*)) ) OR AB ( ((after* OR follow* OR post) N4 (anesthe* OR anaesthe* OR operat* OR surger* OR surgical OR procedure*)) ) OR KW ( ((after* OR follow* OR post) N4 (anesthe* OR anaesthe* OR operat* OR surger* OR surgical OR procedure*)) ) \| Expanders - Apply equivalent subjects Search modes - Find all my search terms \| 30,613 \| \| S44 \| TI ( (postanesthe* OR postanaesthe* OR postoperative* OR postsurger*) ) OR AB ( (postanesthe* OR postanaesthe* OR postoperative* OR postsurger*) ) OR KW ( (postanesthe* OR postanaesthe* OR postoperative* OR postsurger*) ) \| Expanders - Apply equivalent subjects Search modes - Find all my search terms \| 11,131 \| \| S43 \| DE "Postsurgical Complications" \| Expanders - Apply equivalent subjects Search modes - Find all my search terms \| 1,432 \| \| S42 \| S40 OR S41 \| Expanders - Apply equivalent subjects Search modes - Find all my search terms \| 70 \| \| S41 \| TI ( ((postoperative* OR post-operative*) N3 confusion*) ) OR AB ( ((postoperative* OR post-operative*) N3 confusion*) ) OR KW ( ((postoperative* OR post-operative*) N3 confusion*) ) \| Expanders - Apply equivalent subjects Search modes - Find all my search terms \| 40 \| \| S40 \| TI ( ("agitated emergence" OR "emergence agitation*" OR "emergence delirium" OR "emergence excitement*" OR "postanesthe* excitement*" OR "post-anesthe* excitement*" OR "postanaesthe* excitement*" OR "post-anaesthe* excitement*") ) OR AB ( ("agitated emergence" OR "emergence agitation*" OR "emergence delirium" OR "emergence excitement*" OR "postanesthe* excitement*" OR "post-anesthe* excitement*" OR "postanaesthe* excitement*" OR "post-anaesthe* excitement*") ) OR KW ( ("agitated emergence" OR "emergence agitation*" OR "emergence delirium" OR "emergence excitement*" OR "postanesthe* excitement*" OR "post-anesthe* excitement*" OR "postanaesthe* excitement*" OR "post-anaesthe* excitement*") ) \| Expanders - Apply equivalent subjects Search modes - Find all my search terms \| 30 \| \| S39 \| S24 OR S25 OR S26 OR S27 OR S28 OR S29 OR S30 OR S31 OR S32 OR S33 OR S34 OR S35 OR S36 OR S37 OR S38 \| Expanders - Apply equivalent subjects Search modes - Find all my search terms \| 1,111,410 \| \| S38 \| TI ( (logistic N2 (model* OR regression*)) ) OR AB ( (logistic N2 (model* OR regression*)) ) OR KW ( (logistic N2 (model* OR regression*)) ) \| Expanders - Apply equivalent subjects Search modes - Find all my search terms \| 83,822 \| \| S37 \| TI ( (predict* N2 (value* OR variable*)) ) OR AB ( (predict* N2 (value* OR variable*)) ) OR KW ( (predict* N2 (value* OR variable*)) ) \| Expanders - Apply equivalent subjects Search modes - Find all my search terms \| 37,551 \| \| S36 \| TI ( ((anxiet* OR anxious* OR cogni* OR depress* OR geriatric* OR health* OR mental* OR neurocogni* OR neuropsych* OR psych* OR personality) N10 (assess* OR evaluat* OR instrument* OR measure* OR questionnaire* OR psychometric* OR scale* OR score* OR screen* OR survey* OR test*)) ) OR AB ( ((anxiet* OR anxious* OR cogni* OR depress* OR geriatric* OR health* OR mental* OR neurocogni* OR neuropsych* OR psych* OR personality) N10 (assess* OR evaluat* OR instrument* OR measure* OR questionnaire* OR psychometric* OR scale* OR score* OR screen* OR survey* OR test*)) ) OR KW ( ((anxiet* OR anxious* OR cogni* OR depress* OR geriatric* OR health* OR mental* OR neurocogni* OR neuropsych* OR psych* OR personality) N10 (assess* OR evaluat* OR instrument* OR measure* OR questionnaire* OR psychometric* OR scale* OR score* OR screen* OR survey* OR test*)) ) \| Expanders - Apply equivalent subjects Search modes - Find all my search terms \| 848,674 \| \| S35 \| DE "Psychological Assessment" OR DE "Neuropsychological Assessment" OR DE "Halstead Reitan Neuropsychological Battery" OR DE "Luria Nebraska Neuropsychological Battery" OR DE "Mini Mental State Examination" OR DE "Task Switching" OR DE "Wisconsin Card Sorting Test" \| Expanders - Apply equivalent subjects Search modes - Find all my search terms \| 33,757 \| \| S34 \| DE "Psychometrics" OR DE "Classical Test Theory" OR DE "Consistency (Measurement)" OR DE "Error of Measurement" OR DE "External Validity" OR DE "Factor Analysis" OR DE "Internal Validity" OR DE "Item Analysis (Test)" OR DE "Item Response Theory" OR DE "Measurement Invariance" OR DE "Measurement Models" OR DE "Multivariate Analysis" OR DE "Test Construction" OR DE "Test Reliability" OR DE "Test Sensitivity" OR DE "Test Specificity" OR DE "Test Validity" OR DE "Variability Measurement" \| Expanders - Apply equivalent subjects Search modes - Find all my search terms \| 246,939 \| \| S33 \| DE "Personality Measures" \| Expanders - Apply equivalent subjects Search modes - Find all my search terms \| 17,593 \| \| S32 \| DE "Mental Health Screening" \| Expanders - Apply equivalent subjects Search modes - Find all my search terms \| 502 \| \| S31 \| DE "Anxiety Screening" \| Expanders - Apply equivalent subjects Search modes - Find all my search terms \| 36 \| \| S30 \| DE "Depression Screening" \| Expanders - Apply equivalent subjects Search modes - Find all my search terms \| 118 \| \| S29 \| DE "Quality of Life Measures" \| Expanders - Apply equivalent subjects Search modes - Find all my search terms \| 935 \| \| S28 \| DE "Measurement" \| Expanders - Apply equivalent subjects Search modes - Find all my search terms \| 64,549 \| \| S27 \| DE "Risk Assessment" \| Expanders - Apply equivalent subjects Search modes - Find all my search terms \| 29,352 \| \| S26 \| DE "Geriatric Assessment" \| Expanders - Apply equivalent subjects Search modes - Find all my search terms \| 8,037 \| \| S25 \| DE "Mental Status" OR DE "Mini Mental State Examination" \| Expanders - Apply equivalent subjects Search modes - Find all my search terms \| 2,217 \| \| S24 \| DE "Rating Scales" \| Expanders - Apply equivalent subjects Search modes - Find all my search terms \| 26,142 \| \| S23 \| S1 OR S2 OR S3 OR S4 OR S5 OR S6 OR S7 OR S8 OR S9 OR S10 OR S11 OR S12 OR S13 OR S14 OR S15 OR S16 OR S17 OR S18 OR S19 OR S20 OR S21 OR S22 \| Expanders - Apply equivalent subjects Search modes - Find all my search terms \| 1,376,563 \| \| S22 \| TI ( ((preanesthe* OR pre-anesthe* OR preanaesthe* OR pre-anaesthe* OR preoperative* OR pre-operative* OR presurg* OR pre-surg*) N3 cogniti*) ) OR AB ( ((preanesthe* OR pre-anesthe* OR preanaesthe* OR pre-anaesthe* OR preoperative* OR pre-operative* OR presurg* OR pre-surg*) N3 cogniti*) ) OR KW ( ((preanesthe* OR pre-anesthe* OR preanaesthe* OR pre-anaesthe* OR preoperative* OR pre-operative* OR presurg* OR pre-surg*) N3 cogniti*) ) \| Expanders - Apply equivalent subjects Search modes - Find all my search terms \| 225 \| \| S21 \| TI (life N2 satisf*) OR AB (life N2 satisf*) OR KW (life N2 satisf*) \| Expanders - Apply equivalent subjects Search modes - Find all my search terms \| 25,101 \| \| S20 \| TI (expectation* N3 recover*) OR AB (expectation* N3 recover*) OR KW (expectation* N3 recover*) \| Expanders - Apply equivalent subjects Search modes - Find all my search terms \| 376 \| \| S19 \| TI ( (antidepress* OR anxiet* OR anxious* OR depression* OR depressed OR depressive OR fear OR "**locus of control**" OR "mental* outcome*" OR personality OR "personal satisfaction" OR PTSD OR "post-traumatic stress" OR resilien* OR self-efficacy OR "stress immunit*" OR worry*) ) OR AB ( (antidepress* OR anxiet* OR anxious* OR depression* OR depressed OR depressive OR fear OR "locus of control" OR "mental* outcome*" OR personality OR "personal satisfaction" OR PTSD OR "post-traumatic stress" OR resilien* OR self-efficacy OR "stress immunit*" OR worry*) ) OR KW ( (antidepress* OR anxiet* OR anxious* OR depression* OR depressed OR depressive OR fear OR "locus of control" OR "mental* outcome*" OR personality OR "personal satisfaction" OR PTSD OR "post-traumatic stress" OR resilien* OR self-efficacy OR "stress immunit*" OR worry*) ) \| Expanders - Apply equivalent subjects Search modes - Find all my search terms \| 912,065 \| \| S18 \| TI ( (pain N3 (catastrophi* OR perception* OR rumination*)) ) OR AB ( (pain N3 (catastrophi* OR perception* OR rumination*)) ) OR KW ( (pain N3 (catastrophi* OR perception* OR rumination*)) ) \| Expanders - Apply equivalent subjects Search modes - Find all my search terms \| 7,464 \| \| S17 \| TI ( (coping N3 (anticipatory OR approach* OR behavio* OR cognitiv* OR constructive OR primary OR proactive OR skill* OR social OR strateg*)) ) OR AB ( (coping N3 (anticipatory OR approach* OR behavio* OR cognitiv* OR constructive OR primary OR proactive OR skill* OR social OR strateg*)) ) OR KW ( (coping N3 (anticipatory OR approach* OR behavio* OR cognitiv* OR constructive OR primary OR proactive OR skill* OR social OR strateg*)) ) \| Expanders - Apply equivalent subjects Search modes - Find all my search terms \| 50,634 \| \| S16 \| TI ( ((emotion* OR mental) N3 (distress OR health* OR "ill being" OR state* OR symptom* OR stress OR wellbeing OR well-being OR wellness)) ) OR AB ( ((emotion* OR mental) N3 (distress OR health* OR "ill being" OR state* OR symptom* OR stress OR wellbeing OR well-being OR wellness)) ) OR KW ( ((emotion* OR mental) N3 (distress OR health* OR "ill being" OR state* OR symptom* OR stress OR wellbeing OR well-being OR wellness)) ) \| Expanders - Apply equivalent subjects Search modes - Find all my search terms \| 359,279 \| \| S15 \| TI ( ((psycholog* OR psychiatric*) N3 (adaptation* OR condition* OR distress OR factor* OR health* OR "ill being" OR illness* OR symptom* OR status OR stress OR wellbeing OR well-being OR wellness)) ) OR AB ( ((psycholog* OR psychiatric*) N3 (adaptation* OR condition* OR distress OR factor* OR health* OR "ill being" OR illness* OR symptom* OR status OR stress OR wellbeing OR well-being OR wellness)) ) OR KW ( ((psycholog* OR psychiatric*) N3 (adaptation* OR condition* OR distress OR factor* OR health* OR "ill being" OR illness* OR symptom* OR status OR stress OR wellbeing OR well-being OR wellness)) ) \| Expanders - Apply equivalent subjects Search modes - Find all my search terms \| 176,545 \| \| S14 \| DE "Fear" \| Expanders - Apply equivalent subjects Search modes - Find all my search terms \| 32,135 \| \| S13 \| DE "Adaptation" \| Expanders - Apply equivalent subjects Search modes - Find all my search terms \| 11,626 \| \| S12 \| DE "Resilience (Psychological)" \| Expanders - Apply equivalent subjects Search modes - Find all my search terms \| 24,803 \| \| S11 \| DE "Distress" \| Expanders - Apply equivalent subjects Search modes - Find all my search terms \| 32,351 \| \| S10 \| DE "Psychological Stress" \| Expanders - Apply equivalent subjects Search modes - Find all my search terms \| 10,035 \| \| S9 \| DE "Coping Style" \| Expanders - Apply equivalent subjects Search modes - Find all my search terms \| 4,045 \| \| S8 \| DE "Pain Perception" OR DE "Pain Sensitivity" OR DE "Pain Thresholds" \| Expanders - Apply equivalent subjects Search modes - Find all my search terms \| 14,231 \| \| S7 \| DE "Catastrophizing" \| Expanders - Apply equivalent subjects Search modes - Find all my search terms \| 1,262 \| \| S6 \| DE "Posttraumatic Stress Disorder" \| Expanders - Apply equivalent subjects Search modes - Find all my search terms \| 42,886 \| \| S5 \| DE "Personality Traits" OR DE "Personality" \| Expanders - Apply equivalent subjects Search modes - Find all my search terms \| 103,890 \| \| S4 \| DE "Quality of Life" OR DE "Health Related Quality of Life" \| Expanders - Apply equivalent subjects Search modes - Find all my search terms \| 73,087 \| \| S3 \| DE "Mental Health" \| Expanders - Apply equivalent subjects Search modes - Find all my search terms \| 108,987 \| \| S2 \| (DE "Anxiety" OR DE "Anxiety Sensitivity" OR DE "Death Anxiety" OR DE "Health Anxiety" OR DE "Social Anxiety" OR DE "Anxiety Disorders" OR DE "Castration Anxiety" OR DE "Generalized Anxiety Disorder" OR DE "Panic Attack" OR DE "Panic Disorder" OR DE "Phobias" OR DE "Separation Anxiety Disorder" OR DE "Social Anxiety Disorder") \| Expanders - Apply equivalent subjects Search modes - Find all my search terms \| 150,765 \| \| S1 \| DE "Depression (Emotion)" OR DE "Major Depression" OR DE "Dysthymic Disorder" OR DE "Endogenous Depression" OR DE "Late Life Depression" OR DE "Reactive Depression" OR DE "Recurrent Depression" OR DE "Seasonal Affective Disorder" OR DE "Treatment Resistant Depression") \| Expanders - Apply equivalent subjects Search modes - Find all my search terms \| 194,699 \| | |

5. CINAHL

| Interface: **EBSCOhost** - conctent coverage from 1981  Date of Search: 1 Oktober 2024  Number of hits: 1,691 | Field labels   - MH+ = exploded Cinahl Heading - MH = non exploded Cinahl Heading - TI = title - AB = abstract - Nx = within x words, regardless of order - * = truncation of word for alternate endings - ? = 0-1 letter/number - # = 1 letter/number   Note: sometimes “quotation marks” are needed for single search terms to avoid automatic term mapping (lemmatization)1 |
| --- | --- |
| \| **#** \| **Query** \| **Limiters/Expanders** \| **Results** \| \| --- \| --- \| --- \| --- \| \| S51 \| S49 NOT S50 \| Expanders - Apply equivalent subjects Search modes - Find all my search terms \| 1,691 \| \| S50 \| TI ( (child# OR children* OR childhood OR adolescen* OR infant* OR neonat* OR juvenile* OR p#ediatric*) ) NOT TI ( (aged OR aging OR ageing OR female* OR male* OR "middle age*" OR geriatric* OR old OR older* OR elder* OR men* OR man OR senior* OR wom#n*) ) \| Expanders - Apply equivalent subjects Search modes - Find all my search terms \| 587,296 \| \| S49 \| S23 AND S32 AND S48 \| Expanders - Apply equivalent subjects Search modes - Find all my search terms \| 1,778 \| \| S48 \| S35 OR S47 \| Expanders - Apply equivalent subjects Search modes - Find all my search terms \| 14,966 \| \| S47 \| S39 AND S46 \| Expanders - Apply equivalent subjects Search modes - Find all my search terms \| 14,730 \| \| S46 \| S40 OR S41 OR S42 OR S43 OR S44 OR S45 \| Expanders - Apply equivalent subjects Search modes - Find all my search terms \| 219,064 \| \| S45 \| TI ( (delirium OR POCD) ) OR AB ( (delirium OR POCD) ) \| Expanders - Apply equivalent subjects Search modes - Find all my search terms \| 11,116 \| \| S44 \| TI ( ((cogniti* OR neurocogniti* OR neuropsycho* OR neuro-psycho*) N3 (complication* OR change* OR condition* OR decline OR deficit* OR disorder* OR dysfunction* OR failure* OR function* OR impairment* OR outcome* OR recover* OR score* OR state* OR status* OR symptom*)) ) OR AB ( ((cogniti* OR neurocogniti* OR neuropsycho* OR neuro-psycho*) N3 (complication* OR change* OR condition* OR decline OR deficit* OR disorder* OR dysfunction* OR failure* OR function* OR impairment* OR outcome* OR recover* OR score* OR state* OR status* OR symptom*)) ) \| Expanders - Apply equivalent subjects Search modes - Find all my search terms \| 99,338 \| \| S43 \| TI ( (("postoperative complication*" OR "post-operative complication*" ) AND (cogniti* OR neurocogniti* OR neuropsycho* OR neuro-psycho*)) ) OR AB ( (("postoperative complication*" OR "post-operative complication*" ) AND (cogniti* OR neurocogniti* OR neuropsycho* OR neuro-psycho*)) ) \| Expanders - Apply equivalent subjects Search modes - Find all my search terms \| 187 \| \| S42 \| (MH "Recovery+") \| Expanders - Apply equivalent subjects Search modes - Find all my search terms \| 42,112 \| \| S41 \| (MH "Cognition Disorders") OR (MH "Delirium, Dementia, Amnestic, Cognitive Disorders") OR (MH "Delirium") \| Expanders - Apply equivalent subjects Search modes - Find all my search terms \| 43,840 \| \| S40 \| (MH "Cognition+") \| Expanders - Apply equivalent subjects Search modes - Find all my search terms \| 84,865 \| \| S39 \| S36 OR S37 OR S38 \| Expanders - Apply equivalent subjects Search modes - Find all my search terms \| 316,835 \| \| S38 \| TI ( ((after* OR follow* OR post) N4 (anesthe* OR anaesthe* OR operat* OR surger* OR surgical OR procedure*)) ) OR AB ( ((after* OR follow* OR post) N4 (anesthe* OR anaesthe* OR operat* OR surger* OR surgical OR procedure*)) ) \| Expanders - Apply equivalent subjects Search modes - Find all my search terms \| 174,382 \| \| S37 \| TI ( (postanesthe* OR postanaesthe* OR postoperative* OR postsurger*) ) OR AB ( (postanesthe* OR postanaesthe* OR postoperative* OR postsurger*) ) \| Expanders - Apply equivalent subjects Search modes - Find all my search terms \| 134,706 \| \| S36 \| (MH "Postoperative Period") OR (MH "Postoperative Care") OR (MH "Postoperative Complications") \| Expanders - Apply equivalent subjects Search modes - Find all my search terms \| 119,759 \| \| S35 \| S33 OR S34 \| Expanders - Apply equivalent subjects Search modes - Find all my search terms \| 474 \| \| S34 \| TI ( ((postoperative* OR post-operative*) N3 confusion*) ) OR AB ( ((postoperative* OR post-operative*) N3 confusion*) ) \| Expanders - Apply equivalent subjects Search modes - Find all my search terms \| 100 \| \| S33 \| TI ( ("agitated emergence" OR "emergence agitation*" OR "emergence delirium" OR "emergence excitement*" OR "postanesthe* excitement*" OR "post-anesthe* excitement*" OR "postanaesthe* excitement*" OR "post-anaesthe* excitement*") ) OR AB ( ("agitated emergence" OR "emergence agitation*" OR "emergence delirium" OR "emergence excitement*" OR "postanesthe* excitement*" OR "post-anesthe* excitement*" OR "postanaesthe* excitement*" OR "post-anaesthe* excitement*") ) \| Expanders - Apply equivalent subjects Search modes - Find all my search terms \| 374 \| \| S32 \| S24 OR S25 OR S26 OR S27 OR S28 OR S29 OR S30 OR S31 \| Expanders - Apply equivalent subjects Search modes - Find all my search terms \| 991,235 \| \| S31 \| TI ( (logistic N2 (model* OR regression*)) ) OR AB ( (logistic N2 (model* OR regression*)) ) \| Expanders - Apply equivalent subjects Search modes - Find all my search terms \| 159,580 \| \| S30 \| TI ( (predict* N2 (value* OR variable*)) ) OR AB ( (predict* N2 (value* OR variable*)) ) \| Expanders - Apply equivalent subjects Search modes - Find all my search terms \| 53,850 \| \| S29 \| TI ( ((anxiet* OR anxious* OR cogni* OR depress* OR geriatric* OR health* OR mental* OR neurocogni* OR neuropsych* OR psych* OR personality) N10 (assess* OR evaluat* OR instrument* OR measure* OR questionnaire* OR psychometric* OR scale* OR score* OR screen* OR survey* OR test*)) ) OR AB ( ((anxiet* OR anxious* OR cogni* OR depress* OR geriatric* OR health* OR mental* OR neurocogni* OR neuropsych* OR psych* OR personality) N10 (assess* OR evaluat* OR instrument* OR measure* OR questionnaire* OR psychometric* OR scale* OR score* OR screen* OR survey* OR test*)) ) \| Expanders - Apply equivalent subjects Search modes - Find all my search terms \| 566,054 \| \| S28 \| (MH "Predictive Value of Tests") \| Expanders - Apply equivalent subjects Search modes - Find all my search terms \| 58,153 \| \| S27 \| (MH "Risk Assessment") \| Expanders - Apply equivalent subjects Search modes - Find all my search terms \| 172,923 \| \| S26 \| (MH "Geriatric Assessment+") \| Expanders - Apply equivalent subjects Search modes - Find all my search terms \| 18,227 \| \| S25 \| (MH "Mental Status") \| Expanders - Apply equivalent subjects Search modes - Find all my search terms \| 3,618 \| \| S24 \| (MH "Behavior Rating Scales") OR (MH "Psychological Tests+") \| Expanders - Apply equivalent subjects Search modes - Find all my search terms \| 182,731 \| \| S23 \| S1 OR S2 OR S3 OR S4 OR S5 OR S6 OR S7 OR S8 OR S9 OR S10 OR S11 OR S12 OR S13 OR S14 OR S15 OR S16 OR S17 OR S18 OR S19 OR S20 OR S21 OR S22 \| Expanders - Apply equivalent subjects Search modes - Find all my search terms \| 816,596 \| \| S22 \| TI ( ((preanesthe* OR pre-anesthe* OR preanaesthe* OR pre-anaesthe* OR preoperative* OR pre-operative* OR presurg* OR pre-surg*) N3 cogniti*) ) OR AB ( ((preanesthe* OR pre-anesthe* OR preanaesthe* OR pre-anaesthe* OR preoperative* OR pre-operative* OR presurg* OR pre-surg*) N3 cogniti*) ) \| Expanders - Apply equivalent subjects Search modes - Find all my search terms \| 286 \| \| S21 \| TI (life N2 satisf*) OR AB (life N2 satisf*) \| Expanders - Apply equivalent subjects Search modes - Find all my search terms \| 10,436 \| \| S20 \| TI (expectation* N3 recover*) OR AB (expectation* N3 recover*) \| Expanders - Apply equivalent subjects Search modes - Find all my search terms \| 506 \| \| S19 \| TI ( (antidepress* OR anxiet* OR anxious* OR depression* OR depressed OR depressive OR fear OR "locus of control" OR "mental* outcome*" OR personality OR "personal satisfaction" OR PTSD OR "post-traumatic stress" OR resilien* OR self-efficacy OR "stress immunit*" OR worry*) ) OR AB ( (antidepress* OR anxiet* OR anxious* OR depression* OR depressed OR depressive OR fear OR "locus of control" OR "mental* outcome*" OR personality OR "personal satisfaction" OR PTSD OR "post-traumatic stress" OR resilien* OR self-efficacy OR "stress immunit*" OR worry*) ) \| Expanders - Apply equivalent subjects Search modes - Find all my search terms \| 366,725 \| \| S18 \| TI ( (pain N3 (catastrophi* OR perception* OR rumination*)) ) OR AB ( (pain N3 (catastrophi* OR perception* OR rumination*)) ) \| Expanders - Apply equivalent subjects Search modes - Find all my search terms \| 7,317 \| \| S17 \| TI ( (coping N3 (anticipatory OR approach* OR behavio* OR cognitiv* OR constructive OR primary OR proactive OR skill* OR social OR strateg*)) ) OR AB ( (coping N3 (anticipatory OR approach* OR behavio* OR cognitiv* OR constructive OR primary OR proactive OR skill* OR social OR strateg*)) ) \| Expanders - Apply equivalent subjects Search modes - Find all my search terms \| 20,724 \| \| S16 \| TI ( ((emotion* OR mental) N3 (distress OR health* OR "ill being" OR state* OR symptom* OR stress OR wellbeing OR well-being OR wellness)) ) OR AB ( ((emotion* OR mental) N3 (distress OR health* OR "ill being" OR state* OR symptom* OR stress OR wellbeing OR well-being OR wellness)) ) \| Expanders - Apply equivalent subjects Search modes - Find all my search terms \| 188,114 \| \| S15 \| TI ( ((psycholog* OR psychiatric*) N3 (adaptation* OR condition* OR distress OR factor* OR health* OR "ill being" OR illness* OR symptom* OR status OR stress OR wellbeing OR well-being OR wellness)) ) OR AB ( ((psycholog* OR psychiatric*) N3 (adaptation* OR condition* OR distress OR factor* OR health* OR "ill being" OR illness* OR symptom* OR status OR stress OR wellbeing OR well-being OR wellness)) ) \| Expanders - Apply equivalent subjects Search modes - Find all my search terms \| 74,883 \| \| S14 \| (MH "Fear") \| Expanders - Apply equivalent subjects Search modes - Find all my search terms \| 19,279 \| \| S13 \| (MH "Adaptation, Psychological") \| Expanders - Apply equivalent subjects Search modes - Find all my search terms \| 35,813 \| \| S12 \| (MH "Hardiness") \| Expanders - Apply equivalent subjects Search modes - Find all my search terms \| 19,738 \| \| S11 \| (MH "Psychological Distress") \| Expanders - Apply equivalent subjects Search modes - Find all my search terms \| 8,053 \| \| S10 \| (MH "Stress, Psychological") \| Expanders - Apply equivalent subjects Search modes - Find all my search terms \| 63,310 \| \| S9 \| (MH "Coping+") \| Expanders - Apply equivalent subjects Search modes - Find all my search terms \| 45,931 \| \| S8 \| (MH "Self-Efficacy") \| Expanders - Apply equivalent subjects Search modes - Find all my search terms \| 28,645 \| \| S7 \| (MH "Personality") \| Expanders - Apply equivalent subjects Search modes - Find all my search terms \| 14,732 \| \| S6 \| (MH "Stress Disorders, Post-Traumatic") \| Expanders - Apply equivalent subjects Search modes - Find all my search terms \| 28,037 \| \| S5 \| (MH "Personal Satisfaction") \| Expanders - Apply equivalent subjects Search modes - Find all my search terms \| 17,059 \| \| S4 \| (MH "Quality of Life+") \| Expanders - Apply equivalent subjects Search modes - Find all my search terms \| 160,105 \| \| S3 \| (MH "Mental Health") \| Expanders - Apply equivalent subjects Search modes - Find all my search terms \| 63,495 \| \| S2 \| (MH "Anxiety+") OR (MH "Anxiety Disorders+") \| Expanders - Apply equivalent subjects Search modes - Find all my search terms \| 113,585 \| \| S1 \| (MH "Depression+") \| Expanders - Apply equivalent subjects Search modes - Find all my search terms \| 139,921 \| | |

1. Medline

| Interface: **Ovid MEDLINE(R) ALL** content coverage from 1946  Date of Search: May 27, 2025  Number of hits: 3,970  Comment: In Ovid, two or more words are automatically searched as phrases; i.e. no quotation marks are needed | Field labels   - exp/ = exploded MeSH term - / = non exploded MeSH term - .ti,ab,kf. = title, abstract and author keywords - adjx = within x words, regardless of order - * = truncation of word for alternate endings - ? = 0-1 letter/number - # = 1 letter/number |
| --- | --- |
| Database(s): **Ovid MEDLINE(R) ALL**1946 to May 23, 2025 Search Strategy:   \| **#** \| **Searches** \| **Results** \| \| --- \| --- \| --- \| \| 1 \| Depression/ \| 170067 \| \| 2 \| exp Depressive Disorder/ \| 128752 \| \| 3 \| Anxiety/ \| 121363 \| \| 4 \| exp Anxiety Disorders/ \| 96269 \| \| 5 \| Mental Health/ \| 73011 \| \| 6 \| exp "Quality of Life"/ \| 306242 \| \| 7 \| Personal Satisfaction/ \| 26847 \| \| 8 \| Stress Disorders, Post-Traumatic/ \| 45700 \| \| 9 \| Personality/ \| 43680 \| \| 10 \| Self Efficacy/ \| 27174 \| \| 11 \| Catastrophization/ \| 2242 \| \| 12 \| Pain Perception/ \| 3507 \| \| 13 \| Coping Skills/ \| 762 \| \| 14 \| Stress, Psychological/ \| 142552 \| \| 15 \| Psychological Distress/ \| 6018 \| \| 16 \| Resilience, Psychological/ \| 11527 \| \| 17 \| Adaptation, Psychological/ \| 109237 \| \| 18 \| Fear/ \| 41647 \| \| 19 \| ((psycholog* or psychiatric*) adj3 (adaptation* or condition* or distress or factor* or health* or ill being or illness* or symptom* or status or stress or wellbeing or well-being or wellness)).ti,ab,kf. \| 187048 \| \| 20 \| ((emotion* or mental) adj3 (distress or health* or ill being or state* or symptom* or stress or wellbeing or well-being or wellness)).ti,ab,kf. \| 388087 \| \| 21 \| (coping adj3 (anticipatory or approach* or behavio* or cognitiv* or constructive or primary or proactive or skill* or social or strateg*)).ti,ab,kf. \| 35919 \| \| 22 \| (pain adj3 (catastrophi* or perception* or rumination*)).ti,ab,kf. \| 17240 \| \| 23 \| (antidepress* or anxiet* or anxious* or depression* or depressed or depressive or fear or "locus of control" or mental* outcome* or personality or personal satisfaction or PTSD or post-traumatic stress or resilien* or self-efficacy or stress immunit* or worry*).ti,ab,kf. \| 1093514 \| \| 24 \| (expectation* adj3 recover*).ti,ab,kf. \| 737 \| \| 25 \| (life adj2 satisf*).ti,ab,kf. \| 17844 \| \| 26 \| ((preanesthe* or pre-anesthe* or preanaesthe* or pre-anaesthe* or preoperative* or pre-operative* or presurg* or pre-surg*) adj3 cogniti*).ti,ab,kf. \| 794 \| \| 27 \| or/1-26 \| 1910487 \| \| 28 \| Psychiatric Status Rating Scales/ \| 81358 \| \| 29 \| "Mental Status and Dementia Tests"/ \| 3596 \| \| 30 \| Geriatric Assessment/ \| 35128 \| \| 31 \| Risk Assessment/ \| 328223 \| \| 32 \| exp Psychological Tests/ \| 361915 \| \| 33 \| "Predictive Value of Tests"/ \| 233996 \| \| 34 \| Mental Status Schedule/ \| 6290 \| \| 35 \| Psychometrics/ \| 96159 \| \| 36 \| Logistic Models/ \| 156935 \| \| 37 \| ((anxiet* or anxious* or cogni* or depress* or geriatric* or health* or mental* or neurocogni* or neuropsych* or psych* or personality) adj10 (assess* or evaluat* or instrument* or measure* or questionnaire* or psychometric* or scale* or score* or screen* or survey* or test*)).ti,ab,kf. \| 1547534 \| \| 38 \| (predict* adj2 (value* or variable*)).ti,ab,kf. \| 219372 \| \| 39 \| (logistic adj2 (model* or regression*)).ti,ab,kf. \| 510473 \| \| 40 \| or/28-39 \| 2757463 \| \| 41 \| Postoperative Cognitive Complications/ \| 753 \| \| 42 \| Emergence Delirium/ \| 981 \| \| 43 \| (agitated emergence or emergence agitation* or emergence delirium or emergence excitement* or postanesthe* excitement* or post-anesthe* excitement* or postanaesthe* excitement* or post-anaesthe* excitement*).ti,ab,kf. \| 1206 \| \| 44 \| ((postoperative* or post-operative*) adj3 confusion*).ti,ab,kf. \| 242 \| \| 45 \| or/41-44 \| 2793 \| \| 46 \| Postoperative Period/ \| 57820 \| \| 47 \| Postoperative Care/ \| 61361 \| \| 48 \| Postanesthesia Nursing/ \| 1113 \| \| 49 \| Postoperative Complications/ \| 424874 \| \| 50 \| (postanesthe* or postanaesthe* or postoperative* or postsurger*).ti,ab,kf. \| 754879 \| \| 51 \| ((after* or follow* or post) adj4 (anesthe* or anaesthe* or operat* or surger* or surgical or procedure*)).ti,ab,kf. \| 871195 \| \| 52 \| or/46-51 \| 1612488 \| \| 53 \| Cognition/ \| 141160 \| \| 54 \| Cognitive Dysfunction/ \| 47264 \| \| 55 \| Cognition Disorders/ or Neurocognitive Disorders/ \| 77051 \| \| 56 \| Delirium/ \| 13594 \| \| 57 \| "Recovery of Function"/ \| 62986 \| \| 58 \| ((postoperative complication* or post-operative complication*) and (cogniti* or neurocogniti* or neuropsycho* or neuro-psycho*)).ti,ab,kf. \| 829 \| \| 59 \| ((cogniti* or neurocogniti* or neuropsycho* or neuro-psycho*) adj3 (complication* or change* or condition* or decline or deficit* or disorder* or dysfunction* or failure* or function* or impairment* or outcome* or recover* or score* or state* or status* or symptom*)).ti,ab,kf. \| 329197 \| \| 60 \| (delirium or POCD).ti,ab,kf. \| 25915 \| \| 61 \| or/53-60 \| 520072 \| \| 62 \| 52 and 61 \| 37544 \| \| 63 \| 45 or 62 \| 38405 \| \| 64 \| 27 and 40 and 63 \| 4480 \| \| 65 \| (exp Child/ or exp Infant/ or exp Adolescent/ or (child? or children* or childhood or adolescen* or infant* or neonat* or juvenile* or p?ediatric*).ti.) not (exp Aged/ or Middle Aged/ or (aged or aging or ageing or female* or male* or middle age* or geriatric* or old or older* or elder* or men* or man or senior* or wom?n*).ti.) \| 3157673 \| \| 66 \| 64 not 65 \| 4129 \| \| 67 \| 66 not (animals not humans).sh. \| 3970 \| | |

2. Embase

| Interface: **embase.com** content coverage from 1947  Date of Search: May 27, 2025  Number of hits: 4,880  Comment: Emtree is the controlled vocabulary in Embase | Field labels   - /exp = exploded Emtree term - /de = non exploded Emtree term - ti,ab,kw = title, abstract and author keywords - NEAR/x = within x words, regardless of order - * = truncation of word for alternate endings - # = 0-1 letter/number - ? = 1 letter/number |
| --- | --- |
| \| **No.** \| **Query** \| **Results** \| \| --- \| --- \| --- \| \| #71 \| #70 AND ('Article'/it OR 'Article in Press'/it OR 'Conference Paper'/it OR 'Erratum'/it OR 'Preprint'/it OR 'Review'/it OR 'Short Survey'/it) \| 4880 \| \| #70 \| #69 NOT ([animals]/lim NOT [humans]/lim) \| 7309 \| \| #69 \| #61 NOT #68 \| 7613 \| \| #68 \| #64 NOT #67 \| 4154507 \| \| #67 \| #65 OR #66 \| 7385228 \| \| #66 \| aged:ti OR aging:ti OR ageing:ti OR female*:ti OR male*:ti OR 'middle age*':ti OR geriatric*:ti OR old:ti OR older*:ti OR elder*:ti OR men*:ti OR man:ti OR senior*:ti OR wom$n*:ti \| 2141531 \| \| #65 \| 'middle aged'/de OR 'aged'/exp \| 5923740 \| \| #64 \| #62 OR #63 \| 5156759 \| \| #63 \| child$:ti OR children*:ti OR childhood:ti OR adolescen*:ti OR infant*:ti OR neonat*:ti OR juvenile*:ti OR p$ediatric*:ti \| 2211834 \| \| #62 \| 'juvenile'/exp \| 4791382 \| \| #61 \| #26 AND #38 AND #60 \| 8376 \| \| #60 \| #43 OR #59 \| 55177 \| \| #59 \| #49 AND #58 \| 52394 \| \| #58 \| #50 OR #51 OR #52 OR #53 OR #54 OR #55 OR #56 OR #57 \| 845621 \| \| #57 \| delirium:ti,ab,kw OR pocd:ti,ab,kw \| 41092 \| \| #56 \| ((cogniti* OR neurocogniti* OR neuropsycho* OR 'neuro psycho*') NEAR/3 (complication* OR change* OR condition* OR decline OR deficit* OR disorder* OR dysfunction* OR failure* OR function* OR impairment* OR outcome* OR recover* OR score* OR state* OR status* OR symptom*)):ti,ab,kw \| 481245 \| \| #55 \| ('postoperative complication*':ti,ab,kw OR 'post-operative complication*':ti,ab,kw) AND (cogniti*:ti,ab,kw OR neurocogniti*:ti,ab,kw OR neuropsycho*:ti,ab,kw OR 'neuro psycho*':ti,ab,kw) \| 1450 \| \| #54 \| 'convalescence'/de \| 62633 \| \| #53 \| 'delirium'/de \| 42318 \| \| #52 \| 'disorders of higher cerebral function'/de \| 5060 \| \| #51 \| 'cognitive defect'/de \| 256993 \| \| #50 \| 'cognition'/de \| 368725 \| \| #49 \| #44 OR #45 OR #46 OR #47 OR #48 \| 2303938 \| \| #48 \| ((after* OR follow* OR post) NEAR/4 (anesthe* OR anaesthe* OR operat* OR surger* OR surgical OR procedure*)):ti,ab,kw \| 1341202 \| \| #47 \| postanesthe*:ti,ab,kw OR postanaesthe*:ti,ab,kw OR postoperative*:ti,ab,kw OR postsurger*:ti,ab,kw \| 1187664 \| \| #46 \| 'postoperative complication'/de \| 488564 \| \| #45 \| 'postanesthesia nursing'/de \| 971 \| \| #44 \| 'postoperative period'/de OR 'postanesthesia care'/de OR 'postoperative care'/de \| 421581 \| \| #43 \| #39 OR #40 OR #41 OR #42 \| 10405 \| \| #42 \| ((postoperative* OR 'post operative*') NEAR/3 confusion*):ti,ab,kw \| 370 \| \| #41 \| 'agitated emergence':ti,ab,kw OR 'emergence agitation*':ti,ab,kw OR 'emergence delirium':ti,ab,kw OR 'emergence excitement*':ti,ab,kw OR 'postanesthe* excitement*':ti,ab,kw OR 'post-anesthe* excitement*':ti,ab,kw OR 'postanaesthe* excitement*':ti,ab,kw OR 'post-anaesthe* excitement*':ti,ab,kw \| 1749 \| \| #40 \| 'emergence agitation'/de OR 'postoperative delirium'/de \| 7139 \| \| #39 \| 'postoperative cognitive dysfunction'/de \| 2898 \| \| #38 \| #27 OR #28 OR #29 OR #30 OR #31 OR #32 OR #33 OR #34 OR #35 OR #36 OR #37 \| 4041542 \| \| #37 \| (logistic NEAR/2 (model* OR regression*)):ti,ab,kw \| 726389 \| \| #36 \| (predict* NEAR/2 (value* OR variable*)):ti,ab,kw \| 322204 \| \| #35 \| ((anxiet* OR anxious* OR cogni* OR depress* OR geriatric* OR health* OR mental* OR neurocogni* OR neuropsych* OR psych* OR personality) NEAR/10 (assess* OR evaluat* OR instrument* OR measure* OR questionnaire* OR psychometric* OR scale* OR score* OR screen* OR survey* OR test*)):ti,ab,kw \| 2113333 \| \| #34 \| 'statistical model'/de \| 180998 \| \| #33 \| 'psychometry'/exp \| 125893 \| \| #32 \| 'predictive value'/de \| 295350 \| \| #31 \| 'psychologic test'/exp \| 264133 \| \| #30 \| 'risk assessment'/de \| 794164 \| \| #29 \| 'geriatric assessment'/de \| 23873 \| \| #28 \| 'dementia assessment'/exp \| 70189 \| \| #27 \| 'psychological rating scale'/de \| 23211 \| \| #26 \| #1 OR #2 OR #3 OR #4 OR #5 OR #6 OR #7 OR #8 OR #9 OR #10 OR #11 OR #12 OR #13 OR #14 OR #15 OR #16 OR #17 OR #18 OR #19 OR #20 OR #21 OR #22 OR #23 OR #24 OR #25 \| 3200703 \| \| #25 \| ((preanesthe* OR 'pre anesthe*' OR preanaesthe* OR 'pre anaesthe*' OR preoperative* OR 'pre operative*' OR presurg* OR 'pre surg*') NEAR/3 cogniti*):ti,ab,kw \| 1206 \| \| #24 \| (life NEAR/2 satisf*):ti,ab,kw \| 21251 \| \| #23 \| (expectation* NEAR/3 recover*):ti,ab,kw \| 1042 \| \| #22 \| antidepress*:ti,ab,kw OR anxiet*:ti,ab,kw OR anxious*:ti,ab,kw OR depression*:ti,ab,kw OR depressed:ti,ab,kw OR depressive:ti,ab,kw OR fear:ti,ab,kw OR 'locus of control':ti,ab,kw OR 'mental* outcome*':ti,ab,kw OR personality:ti,ab,kw OR 'personal satisfaction':ti,ab,kw OR ptsd:ti,ab,kw OR 'post-traumatic stress':ti,ab,kw OR resilien*:ti,ab,kw OR 'self efficacy':ti,ab,kw OR 'stress immunit*':ti,ab,kw OR worry*:ti,ab,kw \| 1465416 \| \| #21 \| (pain NEAR/3 (catastrophi* OR perception* OR rumination*)):ti,ab,kw \| 24771 \| \| #20 \| (coping NEAR/3 (anticipatory OR approach* OR behavio* OR cognitiv* OR constructive OR primary OR proactive OR skill* OR social OR strateg*)):ti,ab,kw \| 46408 \| \| #19 \| ((emotion* OR mental) NEAR/3 (distress OR health* OR 'ill being' OR state* OR symptom* OR stress OR wellbeing OR 'well being' OR wellness)):ti,ab,kw \| 494864 \| \| #18 \| ((psycholog* OR psychiatric*) NEAR/3 (adaptation* OR condition* OR distress OR factor* OR health* OR 'ill being' OR illness* OR symptom* OR status OR stress OR wellbeing OR 'well being' OR wellness)):ti,ab,kw \| 246307 \| \| #17 \| 'fear'/de \| 92483 \| \| #16 \| 'psychological adjustment'/de \| 5876 \| \| #15 \| 'psychological resilience'/exp \| 15802 \| \| #14 \| 'mental stress'/exp \| 240615 \| \| #13 \| 'coping'/exp \| 95518 \| \| #12 \| 'nociception'/de \| 55196 \| \| #11 \| 'catastrophizing'/de \| 6356 \| \| #10 \| 'self concept'/de \| 126842 \| \| #9 \| 'personality'/de \| 74482 \| \| #8 \| 'posttraumatic stress disorder'/de \| 95232 \| \| #7 \| 'satisfaction'/de OR 'life satisfaction'/de \| 100367 \| \| #6 \| 'wellbeing'/de OR 'emotional well-being'/de \| 101060 \| \| #5 \| 'quality of life'/exp \| 790727 \| \| #4 \| 'mental health'/exp \| 311683 \| \| #3 \| 'anxiety disorder'/exp \| 379495 \| \| #2 \| 'anxiety'/de \| 353193 \| \| #1 \| 'depression'/exp \| 753329 \| | |

3. Web of Science Core Collection

| Interface: **Clarivate Analytics**  Editions and content coverage years= A&HCI - 1975 , ESCI -2019 , SCI-EXPANDED - 1945 , SSCI - 1945  Date of Search: May 27, 2025  Number of hits: 3,244 | Field labels   - TS/Topic = title, abstract, author keywords and Keywords Plus - TI= title - AB = abstract - AK = author keywords - NEAR/x = within x words, regardless of order - * = truncation of word for alternate endings - # = 0-1 letter/number - ? = 1 letter/number   Note: the *Exact search*-function was used for all the searches |
| --- | --- |
| \| **#** \| **Search Query** \| **Results** \| \| --- \| --- \| --- \| \| 1 \| TS=((psycholog* OR psychiatric*) NEAR/3 (adaptation* OR condition* OR distress OR factor* OR health* OR "ill being" OR illness* OR symptom* OR status OR stress OR wellbeing OR well-being OR wellness)) \| 262156 \| \| 2 \| TS=((emotion* OR mental) NEAR/3 (distress OR health* OR "ill being" OR state* OR symptom* OR stress OR wellbeing OR well-being OR wellness)) \| 566057 \| \| 3 \| TS=(coping NEAR/3 (anticipatory OR approach* OR behavio* OR cognitiv* OR constructive OR primary OR proactive OR skill* OR social OR strateg*)) \| 59882 \| \| 4 \| TS=(pain NEAR/3 (catastrophi* OR perception* OR rumination*)) \| 19903 \| \| 5 \| TS=(antidepress* OR anxiet* OR anxious* OR depression* OR depressed OR depressive OR fear OR "locus of control" OR "mental* outcome*" OR personality OR "personal satisfaction" OR PTSD OR "post-traumatic stress" OR resilien* OR self-efficacy OR "stress immunit*" OR worry*) \| 1880670 \| \| 6 \| TS=(expectation* NEAR/3 recover*) \| 1143 \| \| 7 \| TS=(life NEAR/2 satisf*) \| 41469 \| \| 8 \| TS=((preanesthe* OR pre-anesthe* OR preanaesthe* OR pre-anaesthe* OR preoperative* OR pre-operative* OR presurg* OR pre-surg*) NEAR/3 cogniti*) \| 981 \| \| 9 \| #1 OR #2 OR #3 OR #4 OR #5 OR #6 OR #7 OR #8 \| 2374226 \| \| 10 \| TS=((anxiet* OR anxious* OR cogni* OR depress* OR geriatric* OR health* OR mental* OR neurocogni* OR neuropsych* OR psych* OR personality) NEAR/10 (assess* OR evaluat* OR instrument* OR measure* OR questionnaire* OR psychometric* OR scale* OR score* OR screen* OR survey* OR test*)) \| 1837699 \| \| 11 \| TS=(predict* NEAR/2 (value* OR variable*)) \| 318758 \| \| 12 \| TS=(logistic NEAR/2 (model* OR regression*)) \| 559948 \| \| 13 \| #12 OR #11 OR #10 \| 2558779 \| \| 14 \| TS=("agitated emergence" OR "emergence agitation*" OR "emergence delirium" OR "emergence excitement*" OR "postanesthe* excitement*" OR "post-anesthe* excitement*" OR "postanaesthe* excitement*" OR "post-anaesthe* excitement*") \| 1594 \| \| 15 \| TS=((postoperative* OR post-operative*) NEAR/3 confusion*) \| 280 \| \| 16 \| #14 OR #15 \| 1874 \| \| 17 \| TS=(postanesthe* OR postanaesthe* OR postoperative* OR postsurger*) \| 696188 \| \| 18 \| TS=((after* OR follow* OR post) NEAR/4 (anesthe* OR anaesthe* OR operat* OR surger* OR surgical OR procedure*)) \| 898558 \| \| 19 \| #17 OR #18 \| 1349528 \| \| 20 \| TS=(("postoperative complication*" OR "post-operative complication*") AND (cogniti* OR neurocogniti* OR neuropsycho* OR neuro-psycho*)) \| 960 \| \| 21 \| TS=((cogniti* OR neurocogniti* OR neuropsycho* OR neuro-psycho*) NEAR/3 (complication* OR change* OR condition* OR decline OR deficit* OR disorder* OR dysfunction* OR failure* OR function* OR impairment* OR outcome* OR recover* OR score* OR state* OR status* OR symptom*)) \| 439770 \| \| 22 \| TS=(delirium OR POCD) \| 33020 \| \| 23 \| #20 OR #21 OR #22 \| 465043 \| \| 24 \| #23 AND #19 \| 21734 \| \| 25 \| #24 OR #16 \| 22579 \| \| 26 \| #25 AND #13 AND #9 \| 3489 \| \| 27 \| TI=(child$ OR children* OR childhood OR adolescen* OR infant* OR neonat* OR juvenile* OR p$ediatric*) \| 2201960 \| \| 28 \| TI=(aged OR aging OR ageing OR female* OR male* OR "middle age*" OR geriatric* OR old OR older* OR elder* OR men* OR man OR senior* OR wom$n*) \| 2679418 \| \| 29 \| #27 NOT #28 \| 2068839 \| \| 30 \| #26 NOT #29 \| 3244 \| | |

4. PsycINFO

| Interface: **EBSCOhost** - content coverage from 1806  Date of Search: May 27, 2025  Number of hits: 949 | Field labels   - DE = subject heading - TI = title - AB = abstract - KW = author keywords - Nx = within x words, regardless of order - * = truncation of word for alternate endings - ? = 0-1 letter/number - # = 1 letter/number   Note: sometimes “quotation marks” are needed for single search terms to avoid automatic term mapping (lemmatization) |
| --- | --- |
| \| **#** \| **Query** \| **Results** \| \| --- \| --- \| --- \| \| S59 \| S57 NOT S58 \| 949 \| \| S58 \| TI ( (child# OR children* OR childhood OR adolescen* OR infant* OR neonat* OR juvenile* OR p#ediatric*) ) NOT TI ( (aged OR aging OR ageing OR female* OR male* OR "middle age*" OR geriatric* OR old OR older* OR elder* OR men* OR man OR senior* OR wom#n*) ) \| 534,851 \| \| S57 \| S23 AND S39 AND S56 \| 997 \| \| S56 \| S42 OR S55 \| 4,192 \| \| S55 \| S46 AND S54 \| 4,151 \| \| S54 \| S47 OR S48 OR S49 OR S50 OR S51 OR S52 OR S53 \| 291,821 \| \| S53 \| TI ( (delirium OR POCD) ) OR AB ( (delirium OR POCD) ) OR KW ( (delirium OR POCD) ) \| 8,404 \| \| S52 \| TI ( ((cogniti* OR neurocogniti* OR neuropsycho* OR neuro-psycho*) N3 (complication* OR change* OR condition* OR decline OR deficit* OR disorder* OR dysfunction* OR failure* OR function* OR impairment* OR outcome* OR recover* OR score* OR state* OR status* OR symptom*)) ) OR AB ( ((cogniti* OR neurocogniti* OR neuropsycho* OR neuro-psycho*) N3 (complication* OR change* OR condition* OR decline OR deficit* OR disorder* OR dysfunction* OR failure* OR function* OR impairment* OR outcome* OR recover* OR score* OR state* OR status* OR symptom*)) ) OR KW ( ((cogniti* OR neurocogniti* OR neuropsycho* OR neuro-psycho*) N3 (complication* OR change* OR condition* OR decline OR deficit* OR disorder* OR dysfunction* OR failure* OR function* OR impairment* OR outcome* OR recover* OR score* OR state* OR status* OR symptom*)) ) \| 212,949 \| \| S51 \| TI ( (("postoperative complication*" OR "post-operative complication*" ) AND (cogniti* OR neurocogniti* OR neuropsycho* OR neuro-psycho*)) ) OR AB ( (("postoperative complication*" OR "post-operative complication*" ) AND (cogniti* OR neurocogniti* OR neuropsycho* OR neuro-psycho*)) ) OR KW ( (("postoperative complication*" OR "post-operative complication*" ) AND (cogniti* OR neurocogniti* OR neuropsycho* OR neuro-psycho*)) ) \| 84 \| \| S50 \| DE "Neurocognitive Disorders" \| 5,701 \| \| S49 \| DE "Delirium" \| 5,103 \| \| S48 \| DE "Cognitive Impairment" \| 48,949 \| \| S47 \| (DE "Cognition" OR DE "Neurocognition") \| 96,059 \| \| S46 \| S43 OR S44 OR S45 \| 37,960 \| \| S45 \| TI ( ((after* OR follow* OR post) N4 (anesthe* OR anaesthe* OR operat* OR surger* OR surgical OR procedure*)) ) OR AB ( ((after* OR follow* OR post) N4 (anesthe* OR anaesthe* OR operat* OR surger* OR surgical OR procedure*)) ) OR KW ( ((after* OR follow* OR post) N4 (anesthe* OR anaesthe* OR operat* OR surger* OR surgical OR procedure*)) ) \| 31,218 \| \| S44 \| TI ( (postanesthe* OR postanaesthe* OR postoperative* OR postsurger*) ) OR AB ( (postanesthe* OR postanaesthe* OR postoperative* OR postsurger*) ) OR KW ( (postanesthe* OR postanaesthe* OR postoperative* OR postsurger*) ) \| 11,425 \| \| S43 \| DE "Postsurgical Complications" \| 1,493 \| \| S42 \| S40 OR S41 \| 83 \| \| S41 \| TI ( ((postoperative* OR post-operative*) N3 confusion*) ) OR AB ( ((postoperative* OR post-operative*) N3 confusion*) ) OR KW ( ((postoperative* OR post-operative*) N3 confusion*) ) \| 44 \| \| S40 \| TI ( ("agitated emergence" OR "emergence agitation*" OR "emergence delirium" OR "emergence excitement*" OR "postanesthe* excitement*" OR "post-anesthe* excitement*" OR "postanaesthe* excitement*" OR "post-anaesthe* excitement*") ) OR AB ( ("agitated emergence" OR "emergence agitation*" OR "emergence delirium" OR "emergence excitement*" OR "postanesthe* excitement*" OR "post-anesthe* excitement*" OR "postanaesthe* excitement*" OR "post-anaesthe* excitement*") ) OR KW ( ("agitated emergence" OR "emergence agitation*" OR "emergence delirium" OR "emergence excitement*" OR "postanesthe* excitement*" OR "post-anesthe* excitement*" OR "postanaesthe* excitement*" OR "post-anaesthe* excitement*") ) \| 39 \| \| S39 \| S24 OR S25 OR S26 OR S27 OR S28 OR S29 OR S30 OR S31 OR S32 OR S33 OR S34 OR S35 OR S36 OR S37 OR S38 \| 1,144,060 \| \| S38 \| TI ( (logistic N2 (model* OR regression*)) ) OR AB ( (logistic N2 (model* OR regression*)) ) OR KW ( (logistic N2 (model* OR regression*)) ) \| 87,451 \| \| S37 \| TI ( (predict* N2 (value* OR variable*)) ) OR AB ( (predict* N2 (value* OR variable*)) ) OR KW ( (predict* N2 (value* OR variable*)) ) \| 38,706 \| \| S36 \| TI ( ((anxiet* OR anxious* OR cogni* OR depress* OR geriatric* OR health* OR mental* OR neurocogni* OR neuropsych* OR psych* OR personality) N10 (assess* OR evaluat* OR instrument* OR measure* OR questionnaire* OR psychometric* OR scale* OR score* OR screen* OR survey* OR test*)) ) OR AB ( ((anxiet* OR anxious* OR cogni* OR depress* OR geriatric* OR health* OR mental* OR neurocogni* OR neuropsych* OR psych* OR personality) N10 (assess* OR evaluat* OR instrument* OR measure* OR questionnaire* OR psychometric* OR scale* OR score* OR screen* OR survey* OR test*)) ) OR KW ( ((anxiet* OR anxious* OR cogni* OR depress* OR geriatric* OR health* OR mental* OR neurocogni* OR neuropsych* OR psych* OR personality) N10 (assess* OR evaluat* OR instrument* OR measure* OR questionnaire* OR psychometric* OR scale* OR score* OR screen* OR survey* OR test*)) ) \| 877,105 \| \| S35 \| DE "Psychological Assessment" OR DE "Neuropsychological Assessment" OR DE "Halstead Reitan Neuropsychological Battery" OR DE "Luria Nebraska Neuropsychological Battery" OR DE "Mini Mental State Examination" OR DE "Task Switching" OR DE "Wisconsin Card Sorting Test" \| 34,380 \| \| S34 \| DE "Psychometrics" OR DE "Classical Test Theory" OR DE "Consistency (Measurement)" OR DE "Error of Measurement" OR DE "External Validity" OR DE "Factor Analysis" OR DE "Internal Validity" OR DE "Item Analysis (Test)" OR DE "Item Response Theory" OR DE "Measurement Invariance" OR DE "Measurement Models" OR DE "Multivariate Analysis" OR DE "Test Construction" OR DE "Test Reliability" OR DE "Test Sensitivity" OR DE "Test Specificity" OR DE "Test Validity" OR DE "Variability Measurement" \| 252,048 \| \| S33 \| DE "Personality Measures" \| 17,937 \| \| S32 \| DE "Mental Health Screening" \| 561 \| \| S31 \| DE "Anxiety Screening" \| 67 \| \| S30 \| DE "Depression Screening" \| 516 \| \| S29 \| DE "Quality of Life Measures" \| 1,007 \| \| S28 \| DE "Measurement" \| 65,070 \| \| S27 \| DE "Risk Assessment" \| 29,777 \| \| S26 \| DE "Geriatric Assessment" \| 8,087 \| \| S25 \| DE "Mental Status" OR DE "Mini Mental State Examination" \| 2,296 \| \| S24 \| DE "Rating Scales" \| 26,673 \| \| S23 \| S1 OR S2 OR S3 OR S4 OR S5 OR S6 OR S7 OR S8 OR S9 OR S10 OR S11 OR S12 OR S13 OR S14 OR S15 OR S16 OR S17 OR S18 OR S19 OR S20 OR S21 OR S22 \| 1,417,014 \| \| S22 \| TI ( ((preanesthe* OR pre-anesthe* OR preanaesthe* OR pre-anaesthe* OR preoperative* OR pre-operative* OR presurg* OR pre-surg*) N3 cogniti*) ) OR AB ( ((preanesthe* OR pre-anesthe* OR preanaesthe* OR pre-anaesthe* OR preoperative* OR pre-operative* OR presurg* OR pre-surg*) N3 cogniti*) ) OR KW ( ((preanesthe* OR pre-anesthe* OR preanaesthe* OR pre-anaesthe* OR preoperative* OR pre-operative* OR presurg* OR pre-surg*) N3 cogniti*) ) \| 241 \| \| S21 \| TI (life N2 satisf*) OR AB (life N2 satisf*) OR KW (life N2 satisf*) \| 26,003 \| \| S20 \| TI (expectation* N3 recover*) OR AB (expectation* N3 recover*) OR KW (expectation* N3 recover*) \| 383 \| \| S19 \| TI ( (antidepress* OR anxiet* OR anxious* OR depression* OR depressed OR depressive OR fear OR "locus of control" OR "mental* outcome*" OR personality OR "personal satisfaction" OR PTSD OR "post-traumatic stress" OR resilien* OR self-efficacy OR "stress immunit*" OR worry*) ) OR AB ( (antidepress* OR anxiet* OR anxious* OR depression* OR depressed OR depressive OR fear OR "locus of control" OR "mental* outcome*" OR personality OR "personal satisfaction" OR PTSD OR "post-traumatic stress" OR resilien* OR self-efficacy OR "stress immunit*" OR worry*) ) OR KW ( (antidepress* OR anxiet* OR anxious* OR depression* OR depressed OR depressive OR fear OR "locus of control" OR "mental* outcome*" OR personality OR "personal satisfaction" OR PTSD OR "post-traumatic stress" OR resilien* OR self-efficacy OR "stress immunit*" OR worry*) ) \| 935,741 \| \| S18 \| TI ( (pain N3 (catastrophi* OR perception* OR rumination*)) ) OR AB ( (pain N3 (catastrophi* OR perception* OR rumination*)) ) OR KW ( (pain N3 (catastrophi* OR perception* OR rumination*)) ) \| 7,660 \| \| S17 \| TI ( (coping N3 (anticipatory OR approach* OR behavio* OR cognitiv* OR constructive OR primary OR proactive OR skill* OR social OR strateg*)) ) OR AB ( (coping N3 (anticipatory OR approach* OR behavio* OR cognitiv* OR constructive OR primary OR proactive OR skill* OR social OR strateg*)) ) OR KW ( (coping N3 (anticipatory OR approach* OR behavio* OR cognitiv* OR constructive OR primary OR proactive OR skill* OR social OR strateg*)) ) \| 52,392 \| \| S16 \| TI ( ((emotion* OR mental) N3 (distress OR health* OR "ill being" OR state* OR symptom* OR stress OR wellbeing OR well-being OR wellness)) ) OR AB ( ((emotion* OR mental) N3 (distress OR health* OR "ill being" OR state* OR symptom* OR stress OR wellbeing OR well-being OR wellness)) ) OR KW ( ((emotion* OR mental) N3 (distress OR health* OR "ill being" OR state* OR symptom* OR stress OR wellbeing OR well-being OR wellness)) ) \| 378,381 \| \| S15 \| TI ( ((psycholog* OR psychiatric*) N3 (adaptation* OR condition* OR distress OR factor* OR health* OR "ill being" OR illness* OR symptom* OR status OR stress OR wellbeing OR well-being OR wellness)) ) OR AB ( ((psycholog* OR psychiatric*) N3 (adaptation* OR condition* OR distress OR factor* OR health* OR "ill being" OR illness* OR symptom* OR status OR stress OR wellbeing OR well-being OR wellness)) ) OR KW ( ((psycholog* OR psychiatric*) N3 (adaptation* OR condition* OR distress OR factor* OR health* OR "ill being" OR illness* OR symptom* OR status OR stress OR wellbeing OR well-being OR wellness)) ) \| 184,095 \| \| S14 \| DE "Fear" \| 32,832 \| \| S13 \| DE "Adaptation" \| 11,919 \| \| S12 \| DE "Resilience (Psychological)" \| 26,719 \| \| S11 \| DE "Distress" \| 33,928 \| \| S10 \| DE "Psychological Stress" \| 10,164 \| \| S9 \| DE "Coping Style" \| 4,242 \| \| S8 \| DE "Pain Perception" OR DE "Pain Sensitivity" OR DE "Pain Thresholds" \| 14,616 \| \| S7 \| DE "Catastrophizing" \| 1,318 \| \| S6 \| DE "Posttraumatic Stress Disorder" \| 44,330 \| \| S5 \| DE "Personality Traits" OR DE "Personality" \| 105,700 \| \| S4 \| DE "Quality of Life" OR DE "Health Related Quality of Life" \| 75,563 \| \| S3 \| DE "Mental Health" \| 114,203 \| \| S2 \| (DE "Anxiety" OR DE "Anxiety Sensitivity" OR DE "Death Anxiety" OR DE "Health Anxiety" OR DE "Social Anxiety" OR DE "Anxiety Disorders" OR DE "Castration Anxiety" OR DE "Generalized Anxiety Disorder" OR DE "Panic Attack" OR DE "Panic Disorder" OR DE "Phobias" OR DE "Separation Anxiety Disorder" OR DE "Social Anxiety Disorder") \| 155,762 \| \| S1 \| DE "Depression (Emotion)" OR DE "Major Depression" OR DE "Dysthymic Disorder" OR DE "Endogenous Depression" OR DE "Late Life Depression" OR DE "Reactive Depression" OR DE "Recurrent Depression" OR DE "Seasonal Affective Disorder" OR DE "Treatment Resistant Depression") \| 201,375 \| | |

5. CINAHL

| Interface: **EBSCOhost** - conctent coverage from 1981  Date of Search: May 27, 2025  Number of hits: 1,776 | Field labels   - MH+ = exploded Cinahl Heading - MH = non exploded Cinahl Heading - TI = title - AB = abstract - Nx = within x words, regardless of order - * = truncation of word for alternate endings - ? = 0-1 letter/number - # = 1 letter/number   Note: sometimes “quotation marks” are needed for single search terms to avoid automatic term mapping (lemmatization)1 |
| --- | --- |
| \| **#** \| **Query** \| **Results** \| \| --- \| --- \| --- \| \| S51 \| S49 NOT S50 \| 1,776 \| \| S50 \| TI ( (child# OR children* OR childhood OR adolescen* OR infant* OR neonat* OR juvenile* OR p#ediatric*) ) NOT TI ( (aged OR aging OR ageing OR female* OR male* OR "middle age*" OR geriatric* OR old OR older* OR elder* OR men* OR man OR senior* OR wom#n*) ) \| 614,149 \| \| S49 \| S23 AND S32 AND S48 \| 1,869 \| \| S48 \| S35 OR S47 \| 15,682 \| \| S47 \| S39 AND S46 \| 15,434 \| \| S46 \| S40 OR S41 OR S42 OR S43 OR S44 OR S45 \| 230,615 \| \| S45 \| TI ( (delirium OR POCD) ) OR AB ( (delirium OR POCD) ) \| 11,773 \| \| S44 \| TI ( ((cogniti* OR neurocogniti* OR neuropsycho* OR neuro-psycho*) N3 (complication* OR change* OR condition* OR decline OR deficit* OR disorder* OR dysfunction* OR failure* OR function* OR impairment* OR outcome* OR recover* OR score* OR state* OR status* OR symptom*)) ) OR AB ( ((cogniti* OR neurocogniti* OR neuropsycho* OR neuro-psycho*) N3 (complication* OR change* OR condition* OR decline OR deficit* OR disorder* OR dysfunction* OR failure* OR function* OR impairment* OR outcome* OR recover* OR score* OR state* OR status* OR symptom*)) ) \| 104,867 \| \| S43 \| TI ( (("postoperative complication*" OR "post-operative complication*" ) AND (cogniti* OR neurocogniti* OR neuropsycho* OR neuro-psycho*)) ) OR AB ( (("postoperative complication*" OR "post-operative complication*" ) AND (cogniti* OR neurocogniti* OR neuropsycho* OR neuro-psycho*)) ) \| 191 \| \| S42 \| (MH "Recovery+") \| 43,932 \| \| S41 \| (MH "Cognition Disorders") OR (MH "Delirium, Dementia, Amnestic, Cognitive Disorders") OR (MH "Delirium") \| 46,115 \| \| S40 \| (MH "Cognition+") \| 89,896 \| \| S39 \| S36 OR S37 OR S38 \| 322,441 \| \| S38 \| TI ( ((after* OR follow* OR post) N4 (anesthe* OR anaesthe* OR operat* OR surger* OR surgical OR procedure*)) ) OR AB ( ((after* OR follow* OR post) N4 (anesthe* OR anaesthe* OR operat* OR surger* OR surgical OR procedure*)) ) \| 178,603 \| \| S37 \| TI ( (postanesthe* OR postanaesthe* OR postoperative* OR postsurger*) ) OR AB ( (postanesthe* OR postanaesthe* OR postoperative* OR postsurger*) ) \| 138,747 \| \| S36 \| (MH "Postoperative Period") OR (MH "Postoperative Care") OR (MH "Postoperative Complications") \| 120,302 \| \| S35 \| S33 OR S34 \| 504 \| \| S34 \| TI ( ((postoperative* OR post-operative*) N3 confusion*) ) OR AB ( ((postoperative* OR post-operative*) N3 confusion*) ) \| 100 \| \| S33 \| TI ( ("agitated emergence" OR "emergence agitation*" OR "emergence delirium" OR "emergence excitement*" OR "postanesthe* excitement*" OR "post-anesthe* excitement*" OR "postanaesthe* excitement*" OR "post-anaesthe* excitement*") ) OR AB ( ("agitated emergence" OR "emergence agitation*" OR "emergence delirium" OR "emergence excitement*" OR "postanesthe* excitement*" OR "post-anesthe* excitement*" OR "postanaesthe* excitement*" OR "post-anaesthe* excitement*") ) \| 404 \| \| S32 \| S24 OR S25 OR S26 OR S27 OR S28 OR S29 OR S30 OR S31 \| 1,038,290 \| \| S31 \| TI ( (logistic N2 (model* OR regression*)) ) OR AB ( (logistic N2 (model* OR regression*)) ) \| 166,402 \| \| S30 \| TI ( (predict* N2 (value* OR variable*)) ) OR AB ( (predict* N2 (value* OR variable*)) ) \| 55,482 \| \| S29 \| TI ( ((anxiet* OR anxious* OR cogni* OR depress* OR geriatric* OR health* OR mental* OR neurocogni* OR neuropsych* OR psych* OR personality) N10 (assess* OR evaluat* OR instrument* OR measure* OR questionnaire* OR psychometric* OR scale* OR score* OR screen* OR survey* OR test*)) ) OR AB ( ((anxiet* OR anxious* OR cogni* OR depress* OR geriatric* OR health* OR mental* OR neurocogni* OR neuropsych* OR psych* OR personality) N10 (assess* OR evaluat* OR instrument* OR measure* OR questionnaire* OR psychometric* OR scale* OR score* OR screen* OR survey* OR test*)) ) \| 592,836 \| \| S28 \| (MH "Predictive Value of Tests") \| 59,415 \| \| S27 \| (MH "Risk Assessment") \| 188,975 \| \| S26 \| (MH "Geriatric Assessment+") \| 19,627 \| \| S25 \| (MH "Mental Status") \| 3,873 \| \| S24 \| (MH "Behavior Rating Scales") OR (MH "Psychological Tests+") \| 188,972 \| \| S23 \| S1 OR S2 OR S3 OR S4 OR S5 OR S6 OR S7 OR S8 OR S9 OR S10 OR S11 OR S12 OR S13 OR S14 OR S15 OR S16 OR S17 OR S18 OR S19 OR S20 OR S21 OR S22 \| 860,851 \| \| S22 \| TI ( ((preanesthe* OR pre-anesthe* OR preanaesthe* OR pre-anaesthe* OR preoperative* OR pre-operative* OR presurg* OR pre-surg*) N3 cogniti*) ) OR AB ( ((preanesthe* OR pre-anesthe* OR preanaesthe* OR pre-anaesthe* OR preoperative* OR pre-operative* OR presurg* OR pre-surg*) N3 cogniti*) ) \| 297 \| \| S21 \| TI (life N2 satisf*) OR AB (life N2 satisf*) \| 11,301 \| \| S20 \| TI (expectation* N3 recover*) OR AB (expectation* N3 recover*) \| 519 \| \| S19 \| TI ( (antidepress* OR anxiet* OR anxious* OR depression* OR depressed OR depressive OR fear OR "locus of control" OR "mental* outcome*" OR personality OR "personal satisfaction" OR PTSD OR "post-traumatic stress" OR resilien* OR self-efficacy OR "stress immunit*" OR worry*) ) OR AB ( (antidepress* OR anxiet* OR anxious* OR depression* OR depressed OR depressive OR fear OR "locus of control" OR "mental* outcome*" OR personality OR "personal satisfaction" OR PTSD OR "post-traumatic stress" OR resilien* OR self-efficacy OR "stress immunit*" OR worry*) ) \| 387,236 \| \| S18 \| TI ( (pain N3 (catastrophi* OR perception* OR rumination*)) ) OR AB ( (pain N3 (catastrophi* OR perception* OR rumination*)) ) \| 7,555 \| \| S17 \| TI ( (coping N3 (anticipatory OR approach* OR behavio* OR cognitiv* OR constructive OR primary OR proactive OR skill* OR social OR strateg*)) ) OR AB ( (coping N3 (anticipatory OR approach* OR behavio* OR cognitiv* OR constructive OR primary OR proactive OR skill* OR social OR strateg*)) ) \| 22,101 \| \| S16 \| TI ( ((emotion* OR mental) N3 (distress OR health* OR "ill being" OR state* OR symptom* OR stress OR wellbeing OR well-being OR wellness)) ) OR AB ( ((emotion* OR mental) N3 (distress OR health* OR "ill being" OR state* OR symptom* OR stress OR wellbeing OR well-being OR wellness)) ) \| 201,059 \| \| S15 \| TI ( ((psycholog* OR psychiatric*) N3 (adaptation* OR condition* OR distress OR factor* OR health* OR "ill being" OR illness* OR symptom* OR status OR stress OR wellbeing OR well-being OR wellness)) ) OR AB ( ((psycholog* OR psychiatric*) N3 (adaptation* OR condition* OR distress OR factor* OR health* OR "ill being" OR illness* OR symptom* OR status OR stress OR wellbeing OR well-being OR wellness)) ) \| 79,463 \| \| S14 \| (MH "Fear") \| 20,656 \| \| S13 \| (MH "Adaptation, Psychological") \| 37,986 \| \| S12 \| (MH "Hardiness") \| 22,095 \| \| S11 \| (MH "Psychological Distress") \| 9,695 \| \| S10 \| (MH "Stress, Psychological") \| 67,147 \| \| S9 \| (MH "Coping+") \| 48,905 \| \| S8 \| (MH "Self-Efficacy") \| 30,531 \| \| S7 \| (MH "Personality") \| 15,376 \| \| S6 \| (MH "Stress Disorders, Post-Traumatic") \| 29,301 \| \| S5 \| (MH "Personal Satisfaction") \| 18,583 \| \| S4 \| (MH "Quality of Life+") \| 167,374 \| \| S3 \| (MH "Mental Health") \| 69,822 \| \| S2 \| (MH "Anxiety+") OR (MH "Anxiety Disorders+") \| 120,126 \| \| S1 \| (MH "Depression+") \| 147,163 \| | |

**Supplementary Table S2.** Description of exposures and outcomes studied. Data presented as counts and percentage when reported.

| Authors and publication year | *n* | Psychological factor | Instrument used  (psychological) | Cognitive outcome and incidence (%) | Instrument used  (cognitive) | Timepoint outcome assessment |
| --- | --- | --- | --- | --- | --- | --- |
| Liu and colleagues 2023 | 120 | Anxiety | BAI | Delirium: 34 % | CAM | Postoperative days 0,1,3,7 |
| Ren and colleagues, 2021 | 264 | Anxiety | HADS-A | Delirium: 28% | CAM | NR |
| Wang and colleagues, 2025 | 156 | Anxiety  Depression | GAD  PHQ | Delirium: 15.4 % | 3D-CAM | Postoperatively for 7 days, twice daily |
| Leung and colleagues, 2023 | 180 | Anxiety  Depression | HADS-A  GDS | Delirium: 17.8 % | CAM | Postoperative days 1,2,3 |
| Ackenbom and colleagues, 2023 | 183 | Anxiety  Depression | BAI  GDS | Delirium: 12 % | CAM | Postoperative day 1  Follow-up per telephone day 3,5,7 |
| Fukunaga and colleagues, 2022 | 168 | Anxiety  Depression  Personality  Stress | STAI trait + state  GDS  TIPI-J  TAC-24E | Delirium: 15.5 % | DSM-5 criteria | Postoperative days 2-3 |
| de Mul and colleagues, 2022 | 255 | Anxiety  Depression  Stress | HADS-A  GDS  PTSS | Delirium: 17.6 % | CAM, NU-DESC | Postoperative days 1-7, or until discharge, twice daily. |
| Chan and colleagues, 2021 | 199 | Depression | GDS | Delirium: 37 % | DSM 5- criteria, using CAM or DRS-R-98 | Postoperative days 1-5, or until discharge |
| Janssen and colleagues, 2021 | 265 | Depression | CES-D | Delirium: 7.9 % | DOS | NR |
| Rao and colleagues, 2020 | 187 | Depression | GDS | Delirium: 35.8 % | CAM | Every postoperative day until discharge |
| Tao and colleagues, 2019 | 507 | Depression | GDS | Delirium: 23 % | CAM | NR |
| Yamamoto and colleagues, 2016 | 91 | Depression | GDS | Delirium: 26.4 % | CAM | Every postoperative day until day before discharge |
| Cheong and colleagues, 2021 | 447 | Depression | GDS | Delirium: 11 % | CAM  4AT | Postoperative days 1-5, or until discharge |
| Baek and colleagues, 2023 | 91 | Depression | GDS | Delirium: 19.8 % | CAM | NR |
| Radinovic and colleagues, 2014 | 277 | Depression | GDS | Delirium: 31.8 % | CAM | Once every nursing shift during hospital stay |
| Itami and colleagues, 2024 | 255 | Depression | GDS | Delirium: 12.2 % | CAM | Day of surgery until the day before hospital discharge |
| Olofsson and colleagues, 2018 | 135 | Depression | GDS | Delirium: 55.6 % | OBS | 3 times per postoperative day |
| Milisen and colleagues, 2020 | 190 | Anxiety  Depression | APAIS-A, APAIS-NFA  GDS | Delirium: 41 % | CAM-ICU | Postoperative days 1-5 |
| Umoh and colleagues, 2024 | 157 | Depression | GDS | Delirium: 37.6 % | DSM-5 criteria  CAM  DRS-R-98  CDR | Postoperative days 1-5 or until hospital discharge |
| Ackenbom and colleagues, 2021 | 72 | Anxiety  Depression | BAI  GDS | Delirium: 13.8 %  dNCR: 33.3 % | CAM  CVLT-2, BVMT-R, DSST, TRL-A, TRL-B, Stroop | Delirium: Postoperative days 0,1,3,5,7  dNCR: 2 weeks postoperatively |
| Banjongrewadee and colleagues 2020 | 429 | Depression  Personality  Stress | GDS  NI  PSS-10 | Delirium: 5 % | CAM | Every postoperative day, within 72 hours before discharge |
| Dogrul and colleagues, 2020 | 108 | Depression | GDS | Delirium: 3.7 % | 4AT | Postoperative days 3 and 7 |
| Khan and colleagues, 2019 | 234 | Depression | PHQ | Delirium: 9.8 % | DSC | NR |
| Koskderelioglu and colleagues, 2017 | 109 | Depression | BDI | Delirium: 18.3 % | CAM-ICU | Every postoperative day until discharge |
| Shin and colleagues, 2016 | 78 | Anxiety  Depression  Personality | HAS  HRSD  BFI | Delirium: 51 % | CAM  K-DRS | Once every postoperative day 1-7 |
| Mokutani and colleagues, 2016 | 156 | Depression | GDS | Delirium: 31.2 % | CAM | NR |
| Maekawa and colleagues, 2016 | 517 | Depression | GDS | Delirium: 24 % | CAM-ICU | Every postoperative day until discharge |
| Liang and colleagues, 2014 | 232 | Depression | GDS | Delirium: 9 % | CAM | Every postoperative day |
| Tai and colleagues, 2015 | 485 | Depression | GDS | Delirium: 21 % | CAM | NR |
| Deiner and colleagues, 2021 | 167 | Anxiety  Depression | HADS | Delirium: 25.3 %  p-NCD: 12.5 % | CAM  TMT A+B,  DSF/DSB, WAIS, LM I+LM II, CF-A+CF-V, BNT, CVLT | NR for delirium  p-NCD: at 3 months postoperatively |

**Abbreviations:** *3MS* = Modified Mini-Mental State Examination; *BAI* = Beck’s Anxiety Inventory; *BDI* = Beck’s Depression Inventory; *BNT* = Boston Naming Test; *CAM* = Confusion Assessment Method; *CES-D* = Centre for Epidemiologic Studies Depression Scale; *CF-A/V* = Category Fluency – Animals and Vegetables; *CVLT* = California Verbal Learning Test; *dNCR* = Delayed Neurocognitive Recovery; *DOS* = Delirium Observation Screening; *DSC* = Delirium Screening Checklist; *DSF/DSB* = Digit Span Forward and Backward; *DSM-5* = Diagnostic and Statistical Manual of Mental Disorders, Fifth Edition; *GDS* = Geriatric Depression Scale; *DRS-R-98* = Delirium rating scale revised 98; *HADS-A* = Hospital Anxiety and Depression Scale – Anxiety subscale; HRSD = Hamilton Rating Scale For Depression; *LM I / LM II* = Logical Memory Story A – Immediate and Delayed Recall; *MMSE* = Mini-Mental State Examination; *MoCA* = Montreal Cognitive Assessment; *MSET10* = Mental Status Examination Tool – 10 items; *NI* = Neuroticism Inventory; *NR* = not reported *NU-DESC* = Nursing Delirium Screening Scale; *OBS* = Organic Brain Syndrome Scale; *p-NCD* = Postoperative Neurocognitive Disorder; *PHQ*=Patient Health Questionnaire *PSS-10* = 10-Item Perceived Stress Scale; *PTSS* = Posttraumatic Stress Symptoms; *SPMSQ* = Short Portable Mental Status Questionnaire; *STAI* = State-Trait Anxiety Inventory; *TAC-24E* = Tri-Axial Coping Scale – 42-item Extended version; *TICS* = Telephone Interview for Cognitive Status; *TIPI-J* = Ten Item Personality Inventory – Japanese version; *WAIS* = Wechsler Adult Intelligence Scale.

**Supplementary Table S3.** Data results from included studies.

| Authors and publication year | *n* | Available results data for psychological factors associated with postoperative cognitive outcomes | Standardised metric | |  |
| --- | --- | --- | --- | --- | --- |
|  |  |  | **Odds Ratios** | **2-sided p-value** | **Adjusted for** |
| Liu and colleagues 2023 | 120 | Baseline anxiety was an independent risk factor for postoperative delirium, p=0.010 | - | 0.010 |  |
| Ren and colleagues, 2021 | 264 | Anxiety, OR 3.11, 95 % CI [1.14, 8.50], p=0.026 | 3.119 | 0.026 | Age, BMI, ASA, CCI, education, MMSE, anxiety, ICU admission, albumin, serum sodium, blood transfusion, benzo, total fluid |
| Wang and colleagues, 2025 | 156 | Anxiety, OR 0.44, 95 % CI [0.061, 3.191], p=0.418  Depression, p=0.36. Mean scores in delirium and non-delirium groups are not reported. | 0.44 | 0.418  0.36 | Risk factors were randomly combined to establish models  ACCI, PSD, pain, infection |
| Leung and colleagues, 2023 | 180 | Depression, OR 1.14, 95% CI [0.97, 1.35], p=0.11  No differences were seen between groups for characteristics of baseline anxiety measures, p=0.67 | 1.14 | 0.11  0.67 |  |
| Ackenbom and colleagues, 2023 | 183 | Depression OR 1.19, 95% CI [0.98, 1.43], p=0.08  Higher baseline anxiety score in delirium group vs non-delirium group, not statistically significant p=0.34 | 1.19 | 0.08  0.34 | Age, GDS, Education,  FFI, MMSE |
| Fukunaga and colleagues, 2022 | 168 | Personality trait, agreeableness, OR 0.55, 95% CI [0.359, 0.859], p=0.008  Trait anxiety, OR 1.01, 95% CI [0.928, 1.105], p=0.769  State anxiety, OR 0.97, 95% CI [0.89, 1.07], p=0.611  Mean depression scores between groups were similar, and not statistically significant, p=0.905  No group differences in coping styles; one subscale higher in delirium group. | 0.55  1.013  0.976 | 0.008  0.769  0.611  0.905 | Age, STAI, agreeableness, APACHE II score |
| de Mul and colleagues, 2022 | 255 | Delirium group had higher baseline anxiety symptoms, but result was not statistically significant, p=0.083  There was no significant difference in the occurrence of psychopathology. |  | 0.083 |  |
| Chan and colleagues, 2021 | 199 | Depression OR 1.15 95% CI [1.05, 1.26], p=0.003 | 1.13 | 0.003 | Delirium, GDS, demographic, CCI variables |
| Janssen and colleagues, 2021 | 265 | Delirium patients had higher baseline depression symptom score, but result was not statistically significant.  p-values or effect sizes are not reported. |  |  |  |
| Rao and colleagues, 2020 | 187 | Delirium group had higher number of patients with depression, but not statistically significant  p = 0.348 |  | 0.348 |  |
| Tao and colleagues, 2019 | 507 | Delirium group had significantly higher depressive symptom scores.  OR 0.92, 95% CI [0.8, 1.03], p = 0.004 | 0.92 | 0.004 | Known risk variables: all  the CGA components |
| Yamamoto and colleagues, 2016 | 91 | Depression OR 1.3, [95% CI 1.1,1.6], p=0.004 | 1.3 | 0.004 | MMSE, GDS |
| Cheong and colleagues, 2021 | 447 | Depression OR 1.89, 95% CI [0.94, 3.79], p=0.07 | 1.89 | 0.07 | Age, ethnicity, ADL dependency, stroke, electrolyte imbalance, ASA, type of surgery, FFI, MNA, GDS, MOCA |
| Baek and colleagues, 2023 | 91 | Delirium group had higher depression scores, but result was not statistically significant.  P=0.096 |  | 0.096 |  |
| Radinovic and colleagues, 2014 | 277 | 31.8% had delirium alone, 21.7% had an overlap syndrome with depression and delirium.  p-values or effect sizes are not reported. |  |  |  |
| Itami and colleagues, 2024 | 255 | Depression OR 2.5, 95 % CI [0.90, 6.95], p = 0.08 | 2.5 | 0.08 | Exact variables not reported. Multivariate model included variables with p<0.1 in the univariate model. |
| Olofsson and colleagues, 2018 | 135 | Delirium group had higher depression scores vs non-delirium, p=0.004 |  | 0.004 |  |
| Milisen and colleagues, 2020 | 190 | Anxiety OR 0.97, 95% CI [0.88,1.07], p=0.661  Anxiety: need for information OR 0.95, 95% CI [0.83, 1.10], p=0.654  Depression OR 1.04, 95% CI [0.84-1.29]. No p-value reported. | 0.97  0.95  1.04 | 0.661  0.654 | Age, MMSE, GDS, Katz ADL Index, EuroSCORE |
| Umoh and colleagues, 2024 | 157 | Delirium group had significantly higher depressive symptom scores, p = 0.001 |  | 0.001 |  |
| Ackenbom and colleagues, 2021 | 72 | dNCR group had higher baseline anxiety scores, but the result was statistically insignificant, p=0.94  dNCR group had higher baseline depressive symptom scores, but not statistically significant, p=0.05 |  | 0.94  0.05 |  |
| Banjongrewadee and colleagues 2020 | 429 | Depression OR 0.99, 95% CI [0.78-1.27], p=0.991  Stress OR 1.07, 95% CI [0.97, 1.18], p=0.157  Personality trait neuroticism OR 0.98, 95% CI [0.93, 1.04], p=0.583 | 0.99  1.07  0.98 | 0.991  0.157  0.583 | Gender, age, education, alcohol consumption, smoking, history of delirium, history of depression, BMI, NI, MSET10, MoCA, GDS, Barthel Index, PSS-10 |
| Dogrul and colleagues, 2020 | 108 | Delirium group had higher depression scores vs non-delirium group, but not statistically significant, p = 0.460 |  | 0.460 |  |
| Khan and colleagues, 2019 | 234 | Depression OR 0.18  95% CI [0.02, 2.05], p=0.17 | 0.18 | 0.17 | Cognitive deficits, depression, frailty |
| Koskderelioglu and colleagues, 2017 | 109 | Depression OR 1.13, 95% CI [1.05, 1.22], p = 0.002 | 1.13 | 0.002 | Sex |
| Shin and colleagues, 2016 | 78 | Personality neuroticism OR 2.242, 95% CI [1.34,3.75], p=0.00  Personality conscientiousness OR 0.68 95% CI [0.49, 0.96], p = 0.03  Anxiety OR 1.04, 95% CI [0.98, 1.1], p=0.24  Depression OR 1.06 95% CI [0.96, 1.17], p=0.23 | 2.24  0.68  1.04  1.06 | 0.00  0.03  0.24  0.23 |  |
| Mokutani and colleagues, 2016 | 156 | Depression OR 2.56, 95% CI [1.082, 6.213], p=0.033 | 2.565 | 0.033 | Age, gender, BMI, operative time, blood loss, tumor stage, PNI, performance status, ASA, POSSUM |
| Maekawa and colleagues, 2016 | 517 | Depression OR 0.88, 95% CI [0.82, 0.94], P=0.0003 | 0.88 | 0.0003 | Age, sex, surgery type, operative duration, anaesthesia type, transfusion, BMI, serum albumin, Haemoglobin, concentration, serum Creatinine |
| Liang and colleagues, 2014 | 232 | Non-delirium group had higher number of patients with depression vs delirium group. The symptom scores were not statistically associated with delirium, p= 1.00 |  | 1.00 |  |
| Tai and colleagues, 2015 | 485 | Delirium group had significantly higher depressive symptom scores, p=0.020 |  | 0.020 |  |
| Deiner and colleagues, 2021 | 167 | POCD group had higher anxiety and depression scores vs no POCD group, anxiety: p=0.966, depression: p=0.036 |  | 0.966  0.036 |  |

**Blue background** = included in meta-analysis. **Abbreviations**: *AD* = Alzheimer’s Disease; *ADL* = Activities of Daily Living; *APACHE II* = Acute Physiology and Chronic Health Evaluation II; *ASA* = American Society of Anesthesiologists Physical Status Classification System; *Benzo* = Benzodiazepines; *BMI* = Body Mass Index; *CCI* = Charlson Comorbidity Index; *CI* = Confidence Intervals; *CSF* = Cerebrospinal Fluid; *FFI* = Fried Frailty Index; *GDS* = Geriatric Depression Scale; *ICU* = Intensive Care Unit; *MMSE* = Mini-Mental State Examination; *MNA* = Mini Nutritional Assessment; *MoCA* = Montreal Cognitive Assessment; *MSET10* = Mental State Examination Tool – 10 items; *NI* = Neuroticism Inventory; *Ophth* = Ophthalmological; *OR* = Odds Ratios; *PNI* = Prognostic Nutritional Index; *POSSUM* = Physiological and Operative Severity Score for the Enumeration of Mortality and Morbidity; *PSS-10* = 10-item Perceived Stress Scale; *SPMSQ* = Short Portable Mental Status Questionnaire; *STAI* = State-Trait Anxiety Inventory;

**Supplementary Table S4.** Effect direction plot summarizing direction of effects on psychological factors and postoperative cognitive outcomes.


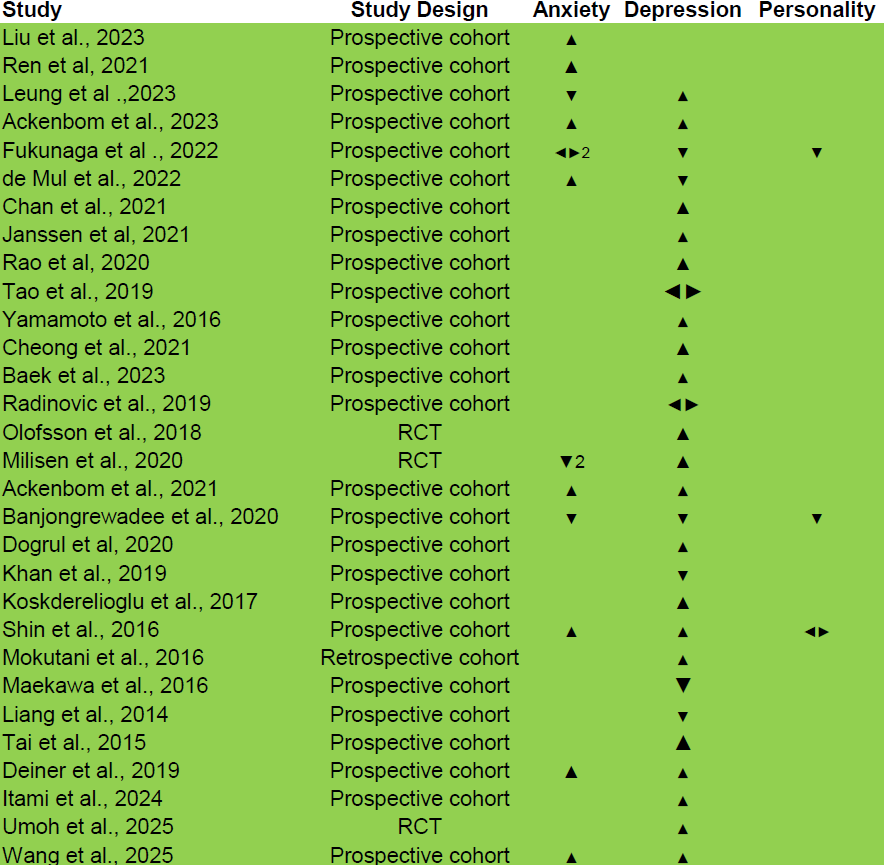


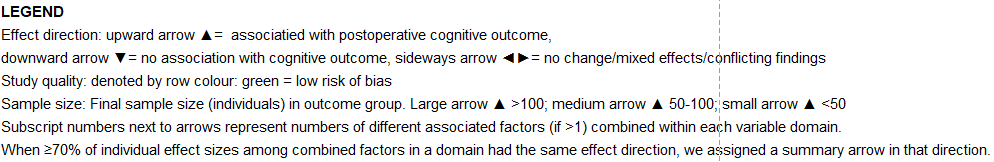


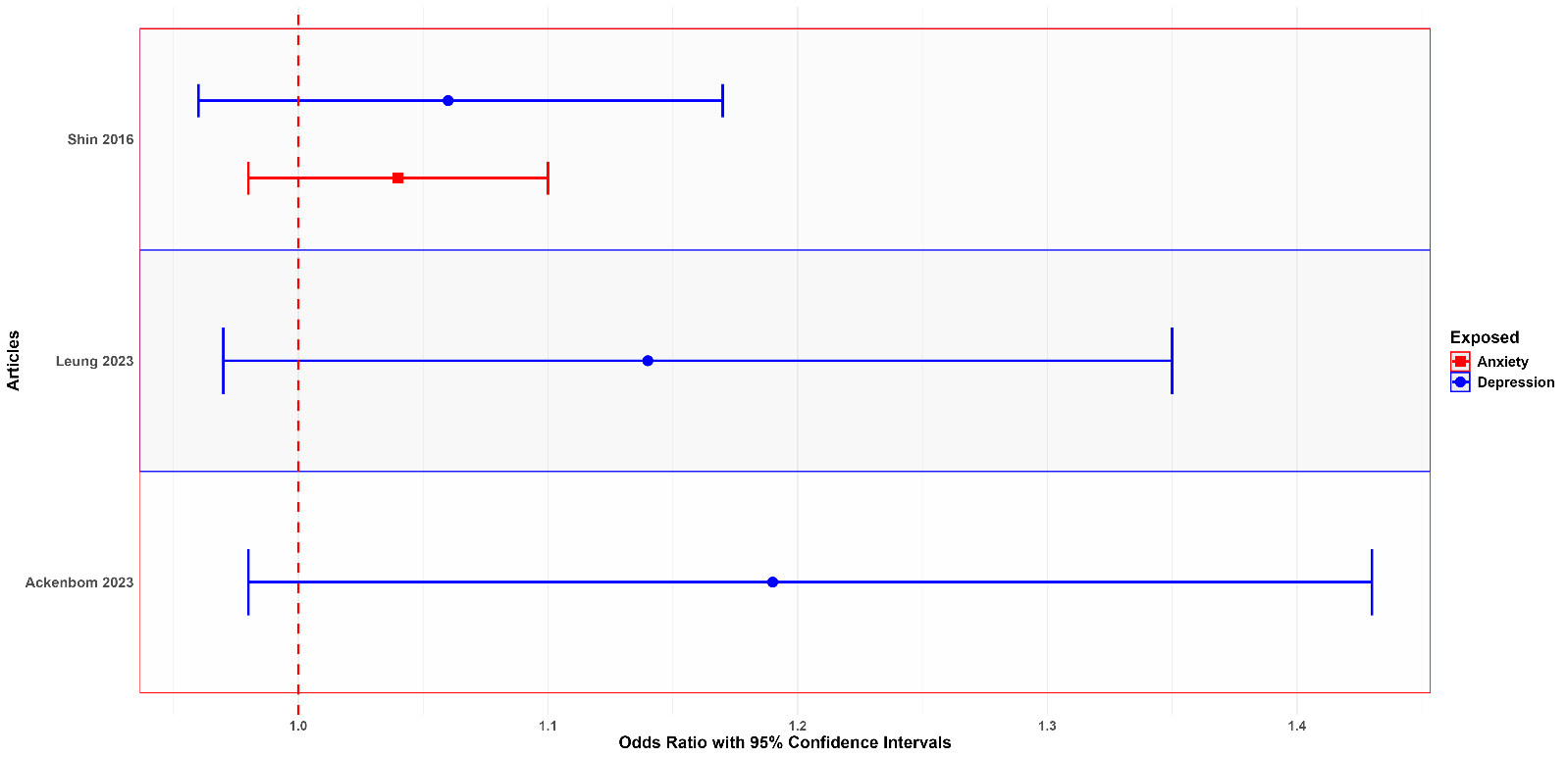


**Supplementary Figure S1.** Forest plot of studies reporting univariate data.

**
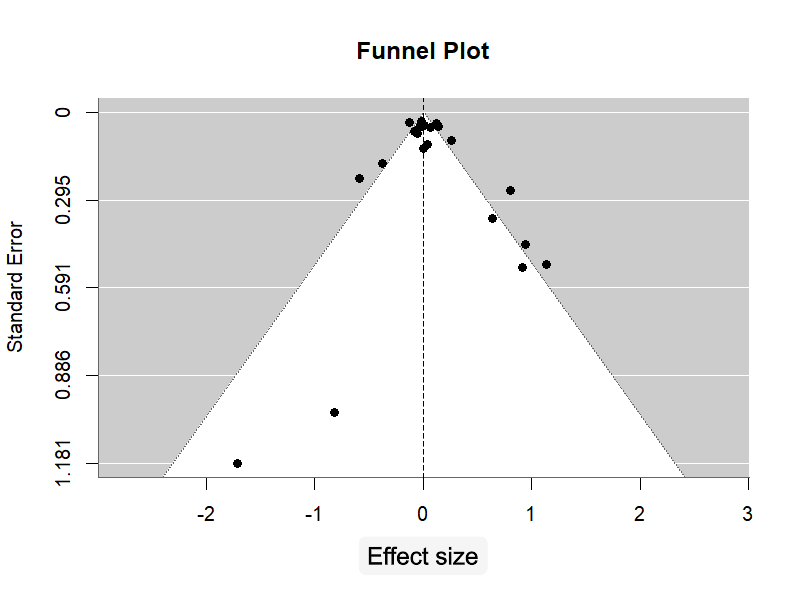
**

**Supplementary Figure S2.** Funnel plot of studies reporting multivariate data.


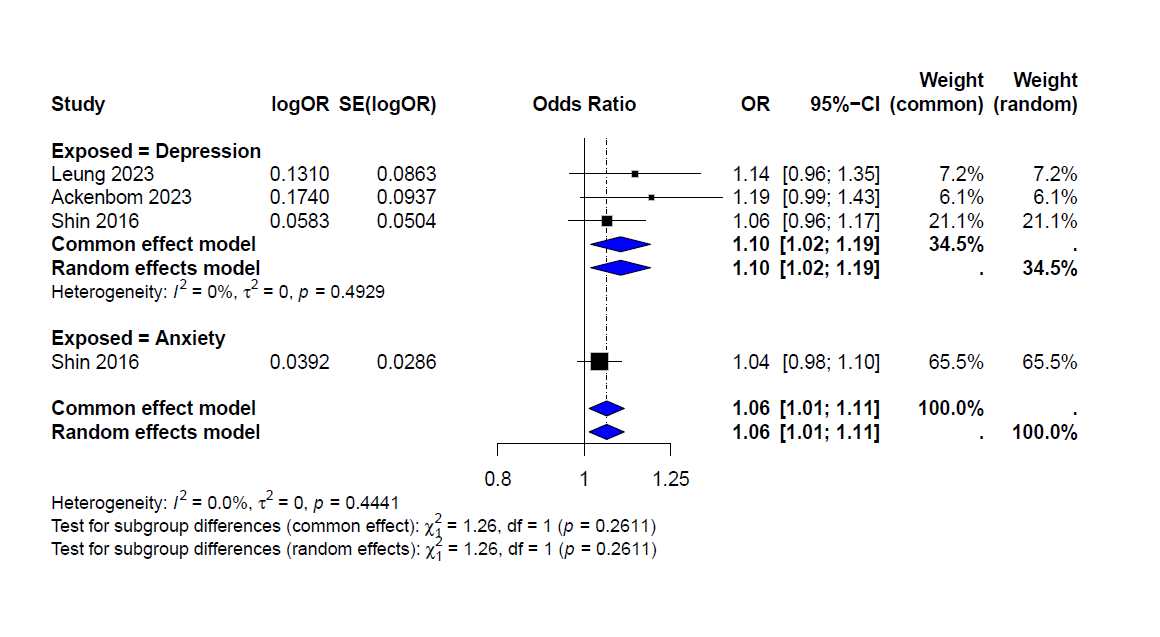


**Supplementary Figure S3.** Random effects model of studies reporting univariate data.
